# A novel point cloud completion model for three-dimensional reconstruction of complex, dynamic population-level crop canopy architecture

**DOI:** 10.1016/j.xplc.2025.101675

**Published:** 2025-12-11

**Authors:** Ziyue Guo, Xin Yang, Yutao Shen, Yang Zhu, Lixi Jiang, Haiyan Cen

**Affiliations:** 1 State Key Laboratory for Vegetation Structure, Function and Construction (VegLab), College of Biosystems Engineering and Food Science, Zhejiang University, Hangzhou 310058, P.R. China; 2 Key Laboratory of Spectroscopy Sensing, Ministry of Agriculture and Rural Affairs, Hangzhou 310058, P.R. China; 3 Zhejiang Key Laboratory of Agricultural Remote Sensing and Information Technology, Hangzhou 310058, P.R. China; 4 Institute of Crop Science, Zhejiang University, Hangzhou 310058, P.R. China

**Keywords:** plant phenotyping, 3D reconstruction, population simulation, point cloud completion, yield estimation

## Abstract

Quantitative characterization of complete canopy architecture is essential for accurate evaluation of crop photosynthesis and yield potential, thereby supporting crop ideotype design. Although various sensing technologies enable three-dimensional (3D) reconstruction of individual plants and canopies, they often fail to describe canopy architecture accurately because of severe occlusion in dense populations. To address this limitation, we developed an effective framework for the 3D reconstruction of complex and dynamic population-scale canopy architecture in rapeseed using unmanned aerial vehicle multi-view imagery combined with a novel point cloud completion model. A complete point cloud generation pipeline was first established to enable automated training data annotation, allowing discrimination between surface points and occluded points within the canopy. The proposed crop population point cloud completion network (CP-PCN) integrates a multi-resolution dynamic graph convolutional encoder, a point pyramid decoder, a dynamic graph convolutional feature extractor, and a generative adversarial network-based loss function to predict occluded canopy points. CP-PCN achieved chamfer distance values of 3.35 to 4.51 cm across four growth stages, outperforming the state-of-the-art transformer-based method PoinTr. Ablation analyses confirmed that each of the four modules contributes to overall model accuracy. In addition, validation experiments showed that the improved architectural completeness achieved by CP-PCN resulted in more accurate yield estimation compared with incomplete and PoinTr-completed point clouds. CP-PCN also demonstrated strong cross-crop generalizability by successfully reconstructing mature rice canopies. Overall, this framework provides a scalable approach for quantitative analysis of complex canopy architectures in field-grown crops.

## Teaser

Accurate three-dimensional reconstruction of dense crop canopies remains challenging because of severe occlusion. This study reports the development of a crop population point cloud completion network (CP-PCN) that effectively recovers occluded canopy structures from unmanned aerial vehicle (UAV) imagery. This approach improves yield estimation accuracy and demonstrates strong generalizability across crop species.

## Introduction

Development of rapeseed (*Brassica napus*) cultivars adapted to dense planting with high yield potential is essential to avoid competition between economic crops and staple food crops for limited arable land. Rational design of crop population canopy architecture is a key strategy to achieve this goal, because the number, size, and spatial arrangement of canopy leaves directly determine light distribution and interception, thereby influencing photosynthetic efficiency and ultimately yield (Liu et al., 2021). In sparse canopies, horizontally oriented leaves maximize light interception, whereas in dense canopies, an optimal architecture consists of upright leaves in the upper layers and progressively more horizontal leaves in deeper layers, which improves overall light use efficiency. In addition, the architectural properties of canopy stems contribute substantially to environmental adaptability. Taller stems are more susceptible to lodging under strong winds, whereas shorter stems may constrain leaf area development and reduce yield potential (Wang et al., 2024). Canopy architecture also shapes the microenvironment within the crop population. Excessively dense canopies limit air circulation and increase the risk of disease, while overly sparse canopies fail to satisfy high-yield requirements (Li et al., 2022b). Despite its importance, the quantitative description of canopy architecture for ideotype design aimed at improving photosynthesis and yield remains challenging, particularly for rapeseed, which exhibits large architectural diversity across the entire growth period.

In recent years, advances in high-throughput plant phenotyping based on three-dimensional (3D) imaging and reconstruction have created new opportunities to characterize crop canopy architecture. Common approaches include depth cameras, laser scanning, or light detection and ranging (LiDAR), and multi-view imaging systems. Depth cameras have been applied in plant phenotyping platforms to acquire 3D data and extract structural traits such as leaf number and leaf area in tomato plants (Masuda, 2021; Watawana and Isaksson, 2024). Hand-held laser scanners have also been used to capture fine-scale 3D point clouds of individual rapeseed plants at maturity, enabling the extraction of traits such as silique volume and length (Ma et al., 2023). However, these approaches depend strongly on sensor-specific imaging conditions and limited detection ranges, which restrict their application mainly to simple targets such as individual plants or greenhouse-grown crops. Active LiDAR systems mounted on unmanned aerial vehicles (UAVs) offer an alternative for large-scale canopy reconstruction at the plot or field level and have achieved high accuracy in extracting maize structural parameters, including plant height with coefficients of determination (R^2^) exceeding 0.97 and leaf area index values of R^2^ = 0.96 (Bailey and Mahaffee, 2017; Jin et al., 2021). Nevertheless, the high cost of LiDAR systems and light absorption at emission wavelengths limit their ability to capture detailed internal canopy features. By contrast, rapid progress in deep learning and multi-view imaging, together with the emergence of advanced methods, such as neural radiance fields (NeRFs), has markedly improved 3D crop reconstruction from multi-view imagery, enabling successful reconstruction in both greenhouse and field environments (Arshad et al., 2024). With the additional RGB color information, multiview RGB imaging-based 3D reconstruction has been widely used to extract a range of canopy structural parameters (Li et al., 2022a; Rossi et al., 2022). However, severe occlusion within dense canopies, especially during late growth stages, still prevents complete reconstruction of canopy architecture, particularly for internal structures.

To overcome these limitations, an ideal approach is to quantitatively describe plant spatial architecture and then use computer graphics techniques to generate virtual crop models with complete canopy structures. Murchie and Burgess (2022) evaluated photosynthetic efficiency of crops under different virtual canopy architectures by modifying leaf distributions, providing a theoretical basis for ideotype design. However, crop growth is influenced by multiple environmental factors, making precise quantification of canopy architectural variation difficult and often resulting in simulations that do not fully represent real field conditions. Several studies have attempted to complete occluded regions at the plant organ level using shape-fitting approaches. Ge et al. (2020) achieved point cloud completion for occluded fruits using predefined topological rules, producing strawberry point clouds with an intersection over union of 0.77 relative to ground truth. Lou et al. (2022) applied biological constraints to complete occluded leaf point clouds, increasing the R^2^ value for projected leaf area estimation from 0.741 to 0.911 and improving total leaf area estimation from 0.338 to 0.964. These approaches depend on limited prior knowledge and explicit structural descriptions, which restrict their applicability to organs with relatively symmetrical architectures.

With the continued development of deep learning, point cloud completion has advanced rapidly, driven by large-scale datasets and improved network architectures. Many publicly available 3D datasets allow random clipping to generate extensive training samples, providing a solid foundation for learning-based completion models (Geiger et al., 2013; Wu et al., 2015; Tchapmi et al., 2019). To handle the unordered and unstructured nature of point clouds, a variety of feature extraction strategies have been proposed, including point-based multi-layer perceptrons (MLPs), graph-based networks, transformer-based models, and voxel-based convolutions (Huang et al., 2020; Xie et al., 2020; Yu et al., 2021; Fei et al., 2022). These methods perform well on public benchmarks dominated by rigid objects such as vehicles and furniture. In agriculture, point cloud completion studies have primarily focused on simpler structures, including fruits or potted plant organs. Magistri et al. (2024) developed a transformer-based model combined with template matching to complete occluded sweet pepper and strawberry point clouds from single-view depth images. Chen et al. (2023) applied the point-based point fractal network (PF-Net) to recover complete leaf structures from top-down depth images of potted cabbage. Similarly, Zhang et al. (2023) integrated a network consisting of multi-scale 3D convolutional neural networks, gridding reverse, cubic feature sampling, and offsetattention module (MSGRNet+OA) into the convolution-based gridding residual network (GRNet) to complete occluded regions of maize leaves scanned by LiDAR. Most existing completion models extract features at fixed spatial scales, limiting their ability to adapt dynamically to architectural changes across growth stages, particularly in complex crop canopies characterized by overlapping leaves and strong spatial heterogeneity. In addition, the lack of accurate and complete population-scale point clouds covering the full growth period remains a major obstacle for training robust completion models.

In this study, we present a comprehensive framework to address 3D reconstruction of complex and dynamic population canopy architecture in field-grown crops. We first developed a virtual–real integration (VRI) simulation approach combined with an occlusion point detection algorithm to generate annotated training datasets containing complete crop population point clouds. We then designed a crop population point cloud completion network (CP-PCN) that integrates a multi-resolution dynamic graph convolutional (MRDG) encoder, a dynamic graph convolutional feature extractor (DGCFE), a point pyramid decoder (PPD), and an adversarial loss to recover occluded canopy structures across growth stages. We further introduced the silique efficiency index (SEI) as a new metric for yield potential estimation and demonstrated that complete point clouds substantially improve yield prediction accuracy. Finally, cross-species validation using rice confirmed that both the completion framework and the dataset generation strategy are transferable to crops with distinct canopy architectures.

## Results

### 3D reconstruction pipeline for the complete architecture of complex, dynamic crop population canopies

**Figure 1:**
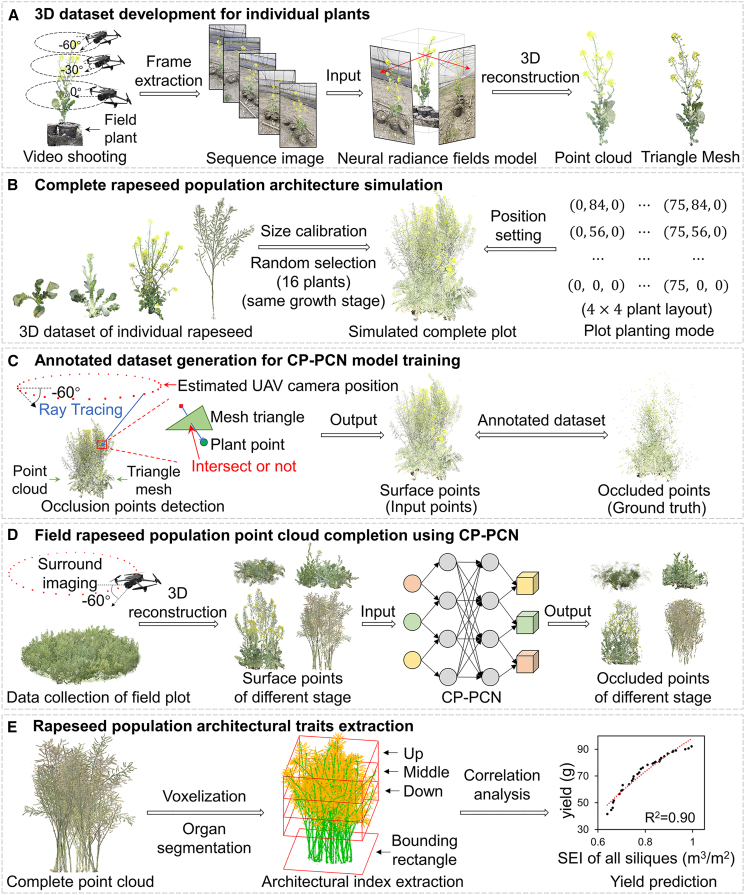
3D reconstruction pipeline for complete architecture of complex, dynamic crop population canopies. **(A)** Development of a 3D dataset for individual rapeseed plants using UAV-based surround filming and neural radiance field (NeRF) reconstruction. **(B)** Construction of simulated population point clouds using the virtual–real integration (VRI) method, which combines 3D models of field-grown individuals with realistic planting patterns. **(C)** Development of an occlusion point detection algorithm to distinguish surface points from occluded points, enabling automated annotation of the training dataset. **(D)** Field data collection from rapeseed populations, with the trained crop population point cloud completion network (CP-PCN) model applied to complete occluded canopy regions. **(E)** Extraction of crop population architectural traits.

**Figure 2:**
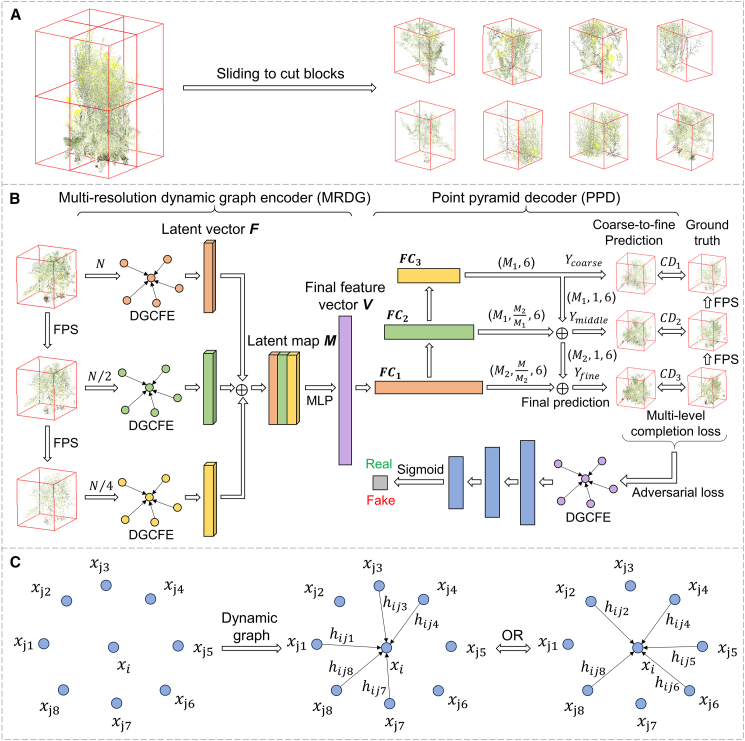
Workflow of the crop population point cloud completion network (CP-PCN). **(A)** The input rapeseed population point cloud is evenly divided into eight blocks using a sliding-window strategy. **(B)** CP-PCN adopts a generative adversarial network (GAN) framework. The generator consists of a multi-resolution dynamic graph encoder (MRDG) and a point pyramid decoder (PPD). Before encoding, the input point cloud is down-sampled into three resolutions using iterative farthest point sampling (FPS). Feature extraction at each resolution is performed using the dynamic graph convolutional feature extractor (DGCFE). **(C)** DGCFE dynamically adjusts feature relationships between the center point and its neighboring points.

As shown in Figure 1, a complete 3D reconstruction pipeline was developed to recover the full canopy architecture of field-grown crops from UAV-acquired multi-view images. The pipeline integrates dataset generation, model training, and trait extraction to enable quantitative analysis of complex and dynamic population architectures. A set of individual rapeseed plants representing four key growth stages was reconstructed using a NeRF model, yielding structurally complete 3D representations suitable for subsequent population-level simulations. Based on these individualplant models, a VRI approach was employed to simulate population plots by arranging plants of the same growth stage under realistic planting densities, thereby generating diverse canopy architectures. An occlusion point detection algorithm was then designed to distinguish surface points from occluded points within the canopy. Here, surface points are defined as 3D coordinates that can be directly reconstructed from UAV multi-view imagery, representing regions visible from at least one camera viewpoint. In contrast, occluded points correspond to canopy regions that remain invisible from all UAV viewpoints due to mutual occlusion within the plant architecture (Long et al., 2020; Macedo and Apolinário, 2023). These datasets enabled supervised training of the proposed CP-PCN, which effectively predicted missing canopy regions from UAV-derived surface point clouds (Figure 2). The reconstructed complete point clouds were subsequently used for canopy trait extraction and yield estimation, providing a reliable foundation for quantitative crop phenotyping.

### Comparison of structural similarity between simulated and real rapeseed population point clouds

**Figure 3:**
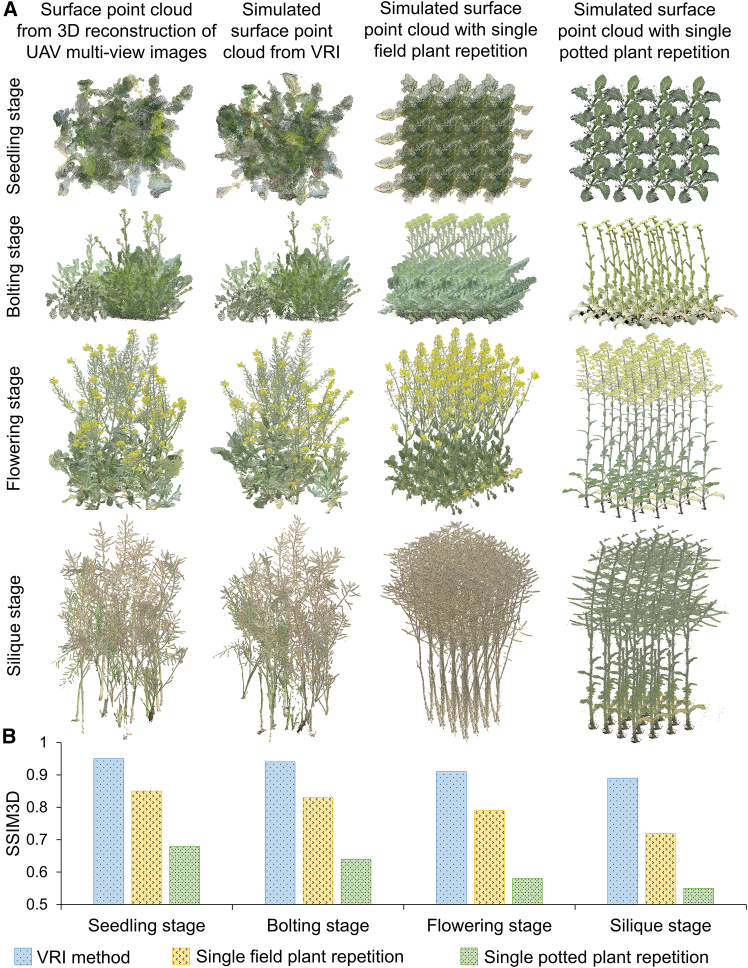
Evaluation of simulated crop population canopy architecture. **(A)** Comparison of canopy architectures across four growth stages using different simulation strategies: the VRI method, single field-grown plant repetition, and single potted plant repetition. **(B)** Quantitative comparison of 3D structural similarity (SSIM3D) between simulated and actual rapeseed population point clouds for each strategy across growth stages.

To validate the reliability and biological realism of the population point clouds generated by the VRI method, we compared simulated canopy architectures with field-reconstructed point clouds at four growth stages. Figures 3A and 3B present qualitative and quantitative evaluations of the simulated crop population canopy architecture using three methods: the proposed VRI method, simple repetition of individual field-grown plants, and simple repetition of individual potted plants. The qualitative results in Figure 3A show that the VRI method closely resembles the field-grown rapeseed populations across all growth stages, with particularly accurate representations during the seedling, bolting, and flowering stages. In contrast, simple repetition of field-grown plants produces less realistic simulations with substantial discrepancies, especially during the silique stage. This method does not capture the structural complexity and diversity inherent in natural rapeseed populations. The repetition of potted plants shows even larger deviations from field conditions, with architectures substantially simpler than those observed in field-grown plants, leading to poor simulation results across all growth stages.

Quantitative results in Figure 3B further support these findings. The 3D structural similarity index (SSIM3D) values for the VRI method were consistently higher than those for the other two methods across all stages. The VRI method achieved SSIM3D values of 0.95, 0.94, 0.91, and 0.89 for the seedling, bolting, flowering, and silique stages, respectively, demonstrating its ability to replicate canopy architectures under field conditions. In contrast, repetition of individual field-grown plants produced moderate similarity scores, with SSIM3D values decreasing from 0.85 at the seedling stage to 0.72 at the silique stage. The potted plant repetition method performed worst, with similarity scores dropping from 0.68 to 0.55 across the same growth stages.

### Evaluation of CP-PCN performance

**Figure 4:**
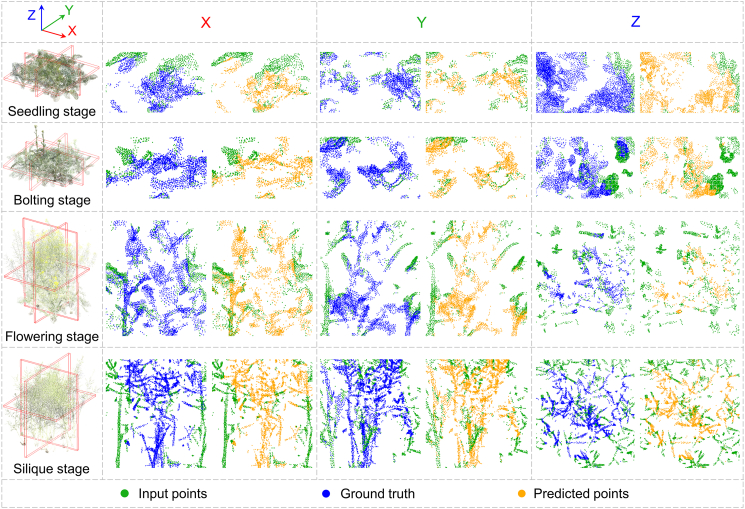
Visualization of complete point clouds at four growth stages generated by CP-PCN. Representative completed point clouds are shown in the *x-*, *y-*, and *z-*plane projections. Input points, ground truth, and predicted points are indicated by green, blue, and orange dots, respectively.

The CP-PCN model demonstrates robust performance in reconstructing rapeseed canopy architecture across four growth stages. As illustrated in Figure 4, the model successfully reconstructs internal canopy architecture, with cross-sectional slices along the *x*, *y*, and *z* planes highlighting detailed internal structures of the completed point clouds. Enlarged views further highlight the model’s ability to recover occluded regions. The full reconstructed point clouds, providing a comprehensive view of canopy architecture, are presented in Supplemental Note 1. At the seedling stage, CP-PCN effectively reconstructed sparse input data, achieving accurate point cloud generation across all spatial dimensions. As canopy complexity increased during the bolting, flowering, and silique stages, the model maintained high reconstruction accuracy, handling increasingly intricate canopy architectures and recovering 3D architectural details with minimal deviation.

**Figure 5:**
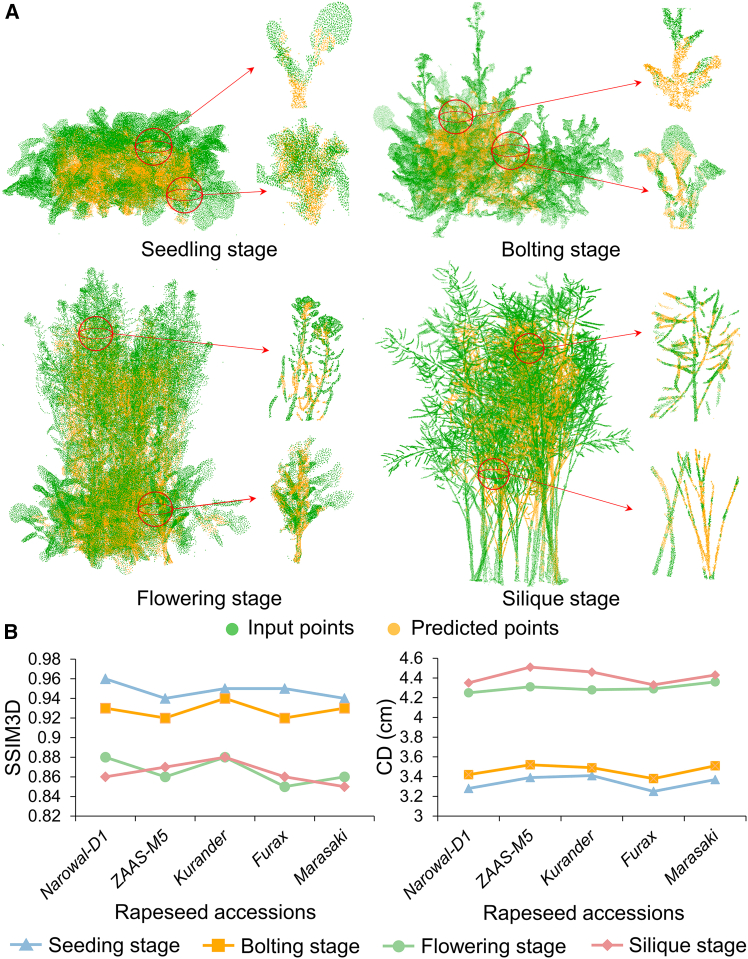
Performance of CP-PCN on field-grown rapeseed populations. **(A)** Point cloud completion for a field-grown rapeseed plot. The incomplete point cloud (green) is completed by CP-PCN (orange), with enlarged views highlighting leaf, flower, silique, and stem architectures. **(B)** Quantitative evaluation of CP-PCN performance across five rapeseed accessions, showing Chamfer distance (CD) and three-dimensional structural similarity index (SSIM3D) values.

To evaluate CP-PCN performance in real-world scenarios, the model was tested using incomplete point clouds collected from field-grown rapeseed populations. As shown in Figure 5A, comparison between the incomplete input data and the completed point clouds generated by CP-PCN illustrates the model’s strong ability to infer missing plant structures, particularly in regions such as the silique and branches. The model effectively addresses occlusions and restores detailed canopy morphology, with predicted point clouds (orange) filling gaps in the original input data (green). Additional completion results from multiple viewing angles and growth stages further demonstrate the model’s consistency and accuracy across perspectives (Supplemental Figures 1–4; Supplemental Video 1).

Supplemental Video 1. Visualization of completed 3D canopy architectures across multiple viewpoints This video presents point cloud completion results generated by CP-PCN at four rapeseed growth stages (seedling, bolting, flowering, and silique). Completed population point clouds are shown from multiple viewing angles to demonstrate structural integrity, continuity, and recovery of occluded canopy regions.

The quantitative evaluation results, presented in Figure 5B, show that CP-PCN achieves consistent performance across all accessions at the four growth stages, as assessed by both Chamfer distance (CD) and SSIM3D. Overall, the model demonstrates high accuracy in predicting canopy architecture, with the average CD and SSIM3D values exhibiting only modest variation across developmental stages. Specifically, at the seedling stage, the average CD was 3.34 cm and the SSIM3D value was 0.95. At the bolting stage, the average CD was 3.46 cm with an SSIM3D value of 0.93. At the flowering stage, the average CD increased to 4.30 cm with an SSIM3D value of 0.87, while at the silique stage, the average CD further increased slightly to 4.42 cm with an SSIM3D value of 0.89. Notably, CP-PCN maintains stable performance across all accessions at the seedling, bolting, flowering, and silique stages, indicating its ability to effectively handle diverse canopy architectures. However, as canopy architectures became more complex at later stages, particularly during flowering and silique formation, a slight decrease in both CD and SSIM3D was observed, reflecting challenges associated with reconstructing more intricate canopy structures. Despite this slight performance degradation, CP-PCN remains robust, confirming its potential for accurate canopy reconstruction across a range of growth stages and plant architectures.

### Comparison with other models

**Figure 6:**
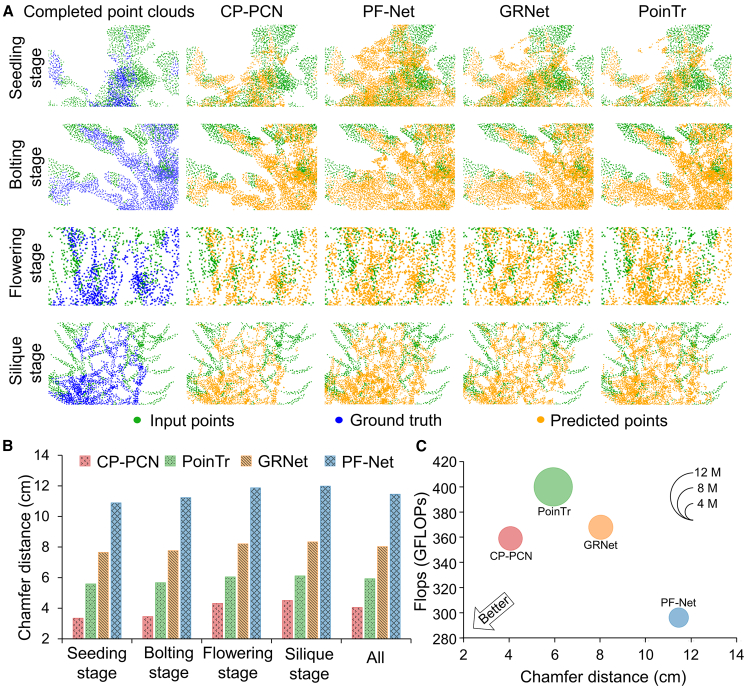
Visualization and comparison of rapeseed population point cloud completion models. **(A)** Visualization of completed point clouds at four growth stages (seedling, bolting, flowering, and silique) generated by CP-PCN, PF-Net, GRNet, and PoinTr. Input points, ground truth, and predicted points are shown in green, blue, and orange dots, respectively. **(B)** Quantitative comparison of Chamfer distance (CD) values for CP-PCN, PF-Net, GRNet, and PoinTr across growth stages. **(C)** Computational comparison of the models in terms of CD and floating-point operations per second (FLOPs), with model size indicated.

Figure 6A presents comparison results of rapeseed point cloud completion across different models. CP-PCN consistently achieved the highest accuracy, capturing fine canopy and silique features with high fidelity and producing 3D reconstructions that closely matched the ground truth. In contrast, PF-Net and GRNet struggled to reconstruct detailed branch architectures and siliques, resulting in substantial discrepancies between the predicted and ground-truth point clouds. Although the PoinTr model performed relatively better at some growth stages, it encountered difficulties in complex canopy formations and often failed to recover critical architectural elements.

Quantitative evaluation further supports these observations (Figure 6B). CP-PCN outperformed all other models across all growth stages, with CD values of 3.35, 3.46, 4.32, and 4.51 cm at the seedling, bolting, flowering, and silique stages, respectively, yielding an overall average CD of 4.05 cm. In contrast, PF-Net and GRNet exhibited higher CD values, indicating weaker reconstruction performance, particularly at later growth stages. At the silique stage, the CD values for PF-Net and GRNet reached 11.98 and 8.34 cm, respectively. Although PoinTr achieved improved accuracy relative to PF-Net and GRNet, its CD values remain consistently higher than those of CP-PCN.

In addition to reconstruction accuracy, computational efficiency and model size of each algorithm were evaluated (Figure 6C). CP-PCN achieved a favorable balance between high accuracy and computational efficiency, making it well suited for large-scale, high-throughput field applications. In contrast, PF-Net was computationally efficient due to its simplicity, but its poor reconstruction performance limits its applicability to high-precision tasks. PoinTr and GRNet, while achieving relatively higher accuracy, incurred substantially greater computational costs and larger model sizes. Specifically, PoinTr requires approximately 420 giga–floating-point operations per second, resulting in longer inference times.

### Estimation of rapeseed yield

**Figure 7:**
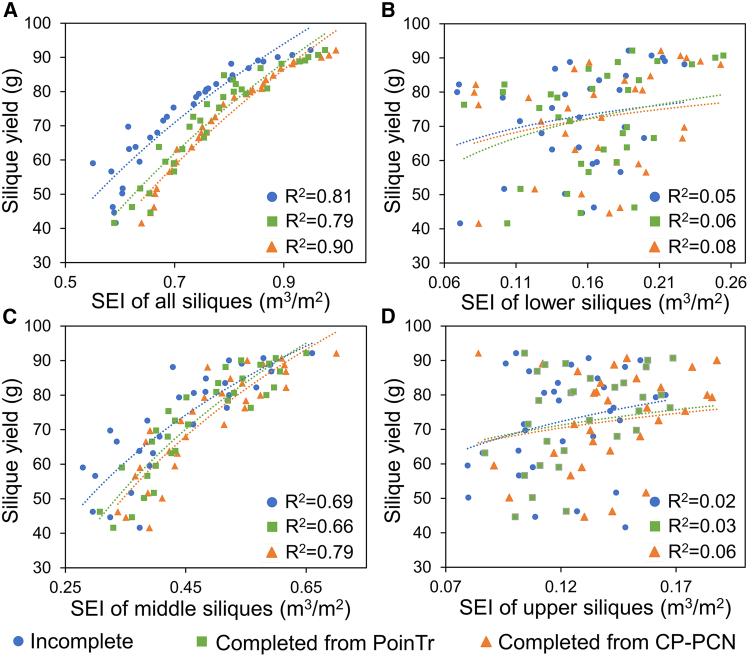
Regression analysis between the silique efficiency index (SEI) and measured silique yield based on incomplete and completed point clouds. Comparison of yield prediction performance based on the silique efficiency index (SEI) derived from incomplete point clouds, point clouds completed by PoinTr, and point clouds completed by CP-PCN. SEI was calculated using all siliques **(A)**, lower-canopy siliques **(B)**, middle-canopy siliques **(C)**, and upper-canopy siliques **(D)**.

The SEI derived from CP-PCN-based completed point clouds exhibits the strongest correlation with rapeseed yield among all datasets (Figure 7). Regression analysis shows that SEI calculated from the completed point clouds achieved an R^2^ of 0.90, compared with 0.81 for incomplete point clouds and 0.79 for PoinTr-based completed point clouds. These results indicate that CP-PCN not only restores missing canopy architecture more effectively but also improves the biological interpretability of reconstructed traits for yield prediction.

Among the different canopy layers, SEI in the middle silique zone displays the highest correlation with yield, consistent with previous findings that siliques located in the middle canopy contribute most significantly to crop productivity (Lin et al., 2024). In contrast, SEI values from the upper and lower canopy layers show weak correlations (R^2^ < 0.10), likely due to uneven spatial silique distribution and mutual shading effects within dense canopy architectures.

## Discussion

### Importance of the high-quality annotated data for modeling

The quality of the training dataset is a critical determinant of deep learning model performance. A high-quality dataset must be representative, covering a broad range of target scenarios, sufficiently diverse to reflect relevant states and conditions, and precisely annotated to prevent the model from learning biased or erroneous patterns (Paullada et al., 2021). In constructing the rapeseed population point cloud completion dataset, we first introduced a novel simulation method, termed VRI, which generated more realistic simulations by explicitly capturing the dynamic nature of rapeseed canopy architectures throughout development, compared with conventional approaches based on replicating individual plant data. During the seedling stage, canopy architectures of different individuals within the same accession are relatively uniform, making traditional replication approaches reasonably effective (Sun et al., 2021). However, as plants progress to the flowering and silique stages, substantial variability in canopy architecture emerges even among individuals of the same accession, and simple replication of individual plants fails to represent this diversity at the population level. In addition, the proposed occlusion point detection algorithm successfully identified truly occluded points from simulated complete canopy point clouds, which are essential targets for model learning in the training dataset. This strategy is consistent with the principles of multi-view 3D reconstruction, where UAV-based imaging primarily captures surface information while internal canopy structures remain occluded and unreconstructed. Existing approaches often generate training data by randomly clipping complete point clouds, leading to many surface points being incorrectly treated as missing regions, which ultimately reduces completion accuracy (Huang et al., 2020; Chen et al., 2023). Furthermore, as shown in Supplemental Note 2, quantitative analysis demonstrates that models trained with visibility-based occlusion labeling achieved substantially lower CD values across all four growth stages than those trained using random clipping, confirming that realistic missing-region patterns and accurate occlusion annotation substantially improve learning efficiency and generalization performance.

### Effects of network components and hyperparameters on CP-PCN performance

Compared with single-plant completion, population-level canopy completion poses greater challenges because missing regions mainly arise from inter-plant occlusions and spatial interactions within dense plots, and the model must reconstruct thin organs while avoiding structurally implausible hallucinations. Ablation results presented in Supplemental Note 3 indicate that all four network components contribute to overall performance, although their functional roles differ. The DGCFE provided the largest performance gain among single-module configurations, consistent with the ability of dynamic graph convolution to learn local geometric relationships by updating neighborhoods in feature space, which is particularly effective for capturing irregular organ boundaries and occlusion-induced discontinuities in plant point clouds (Zhang et al., 2021). The MRDG further enhanced robustness by encoding geometric information at multiple resolutions, enabling the network to represent both plot-scale organization and organ-scale details across developmental stages (Huang et al., 2020). In contrast, the most pronounced additional gains in stronger model configurations originated from the PPD and the generative adversarial network (GAN)-based loss. The PPD refines predictions hierarchically and preserves multi-level features during decoding, facilitating the recovery of fine-scale structures such as slender siliques and panicles that are often oversmoothed in coarse reconstructions (Li et al., 2024). The GAN-based loss introduces distribution-level regularization that promotes globally coherent and realistic point distributions, suppressing scattered artifacts that can otherwise distort downstream volumetric trait estimation in dense canopies (Sarmad et al., 2019). Collectively, these results indicate that MRDG and DGCFE primarily enhance structural feature encoding, whereas PPD and GAN-based supervision are critical for realistic and fine-grained recovery, with their combined use yielding the highest completion accuracy across growth stages.

Beyond the ablation study, a sensitivity analysis of key hyperparameters, including the number of nearest neighbors (*k*), the number of encoder layers (*O*), and the input and output point cloud sizes (*N* and *M*), provided additional insight into CP-PCN performance (Supplemental Figure 5). Model performance stabilized when *k exceeded* 20, indicating that this setting effectively captures spatial relationships without introducing unnecessary computational overhead. Similarly, *O equal to* five layers offered an optimal balance between representational depth and computational efficiency. Shallower configurations limited the extraction of high-level features, whereas deeper networks increased model complexity without measurable gains in accuracy. Moreover, increasing *N* and *M* beyond 8192 did not yield substantial improvements in reconstruction accuracy. Instead, larger point clouds primarily increased point density, which did not improve the network’s ability to resolve structural details. Together, these results indicate that CP-PCN operates efficiently with moderate point cloud sizes and that careful hyperparameter selection is essential for achieving both high accuracy and computational efficiency.

### Applicability and scalability of the CP-PCN framework

The robustness and applicability of the CP-PCN framework were systematically evaluated under varying planting densities, occlusion levels, and imaging conditions. The accuracy of UAV-based reconstruction is strongly influenced by canopy occlusion, which is itself dependent on plant spacing. To examine this relationship, point cloud datasets were generated under different planting configurations. As summarized in Supplemental Note 4, CP-PCN maintained high completion accuracy when the average missing ratio remained below 50%, corresponding to inter-plant spacing greater than 20 cm, with average CD values below 6 cm. When occlusion exceeded this threshold, completion accuracy declined sharply, indicating that severe data loss restricts the availability of structural cues required for reliable reconstruction. This degradation primarily reflects the fundamental limitation that self-occluded canopy regions cannot be observed by UAV imagery, leading to missing information that current models cannot infer. In addition, reconstruction completeness was influenced by plot size relative to the camera’s effective field of view. For the UAV system used in this study, breeding plots with both length and width smaller than approximately 4 m achieved optimal multi-view coverage, whereas larger plots often exhibited incomplete reconstruction at plot edges due to insufficient imaging overlap.

The computational efficiency of CP-PCN was further evaluated to assess its suitability for high-throughput phenotyping. As detailed in Supplemental Note 5, the complete workflow from model construction to application was quantitatively analyzed. The model construction phase, encompassing image acquisition, pose estimation, NeRF reconstruction, dataset generation, and network training, required approximately 15 h. After training, the application phase was highly efficient, requiring roughly 20 s to process a complete field plot, with only 2 s devoted to model inference. These results demonstrate that CP-PCN achieves a practical balance between reconstruction accuracy and computational cost, supporting its deployment in large-scale, multi-plot phenotyping studies.

To further assess cross-crop generalizability, CP-PCN was retrained from scratch using a rice completion dataset generated with the same VRI pipeline. Despite the markedly different canopy architectures of rice compared with rapeseed, CP-PCN successfully reconstructed occluded rice canopy regions and achieved completion accuracy comparable to that observed for rapeseed (CD = 3.86 cm; SSIM3D = 0.91). These findings indicate that CP-PCN learns geometry-driven representations of plant architecture and can be extended to other crops using the same dataset generation strategy. Detailed experimental procedures, visualizations, and quantitative evaluations are provided in Supplemental Note 6.

### Contribution of CP-PCN to crop population canopy analysis

The CP-PCN model presented in this study provides an effective approach for obtaining complete architectural information at the crop population level. Validation using field-grown rapeseed populations demonstrated that point clouds completed by CP-PCN substantially outperformed incomplete point clouds with respect to yield estimation accuracy. Conventional rapeseed yield estimation relies on silique-related traits such as silique number, length, or weight, which are typically measured without accounting for the spatial distribution of siliques within the canopy (Zheng et al., 2022). However, pronounced 3D heterogeneity and stage-dependent architectural variation in rapeseed canopies complicate yield estimation based on such simplified descriptors. By generating complete population-scale point clouds, CP-PCN enables a more faithful representation of true canopy structure and supports spatially resolved quantification of silique distribution.

In our study, the canopy was divided into upper, middle, and lower layers, and the contribution of each layer to yield was quantified. The SEI of the middle silique layer showed the strongest correlation with overall yield, reflecting both higher silique volume and more efficient light interception relative to the upper and lower layers. Importantly, SEI derived from CP-PCN-completed point clouds exhibited stronger yield correlations than SEI derived from either incomplete point clouds or PoinTr-completed point clouds. This difference is primarily attributable to the quality of silique-region reconstruction. In this framework, SEI is calculated using voxel-based silique volume estimation, which is sensitive to both missing silique structures and completion-induced artifacts. We observed that PoinTr can introduce irregular silique-like outliers around pod clusters, artificially inflating occupied voxel counts and weakening the structural signal associated with true silique volume, in some cases producing correlations even lower than those obtained from incomplete point clouds. In contrast, CP-PCN is explicitly designed to preserve geometric integrity in complex crop canopies through local geometry modeling, multi-scale feature aggregation, and adversarial regularization. The graph-based feature extraction modules (MRDG and DGCFE) capture local neighborhood relationships and edge-level geometry, which are well suited for representing slender, clustered silique structures and their spatial continuity under occlusion. The PPD aggregates multi-resolution features to refine local details while maintaining coherent global canopy organization. In addition, the GAN-based loss imposes a structural prior that discourages unrealistic scattered point distributions, an approach widely adopted to improve plausibility and smoothness in 3D completion and reconstruction tasks (Fei et al., 2022). Together, these design elements enable CP-PCN to recover occluded internal siliques while minimizing completion artifacts, resulting in more reliable SEI estimates and a stronger, biologically meaningful relationship with yield.

Finally, CP-PCN enables the quantification of architectural traits that were previously inaccessible, supporting their use as digital agronomic traits for machine-based analysis of canopy formation and development. These traits can be integrated with genomic data to identify loci associated with canopy architecture and productivity, opening new avenues for large-scale genetic improvement and breeding selection based on population-level structural characteristics. Additional evaluation of CP-PCN on other canopy structural traits, such as leaf area index, further supports the broad applicability of canopy completion for crop phenotyping (Supplemental Note 7). Future integration of individual plant- or organ-level semantic segmentation into the completion pipeline would allow simultaneous completion and structural attribution, further enhancing the extraction of organ-specific and plant-specific architectural traits from population point clouds.

## Methods

### Experimental design and data acquisition

Two experimental setups were established for data acquisition: a field experiment and a greenhouse potted plant experiment. The field experiment was designed to capture 3D models of individual plants and surface point clouds of rapeseed populations under natural field conditions. The potted-plant experiment aimed to assess differences in canopy architecture between plants grown under controlled and field environments. Field experiments were conducted at the Jiaxing Academy of Agricultural Sciences, Zhejiang Province, China (120°41′39′′E, 30°51′14′′N). A total of 300 rapeseed accessions were planted with three replicates, resulting in 900 experimental plots (Supplemental Figure 6). Each plot contained 16 plants arranged with 28 cm row spacing and 25 cm plant spacing. Uniform growth conditions were maintained through optimized water and nutrient management. Additionally, four rapeseed accessions with distinct canopy architectures were randomly selected for potted cultivation at the Zhejiang University Agricultural Experimental Station, Hangzhou, China (120°4′46′′E, 30°18′31′′N).

For field-grown individual plants, one plant was randomly selected from the center of each of 100 plots (chosen from one replicate of the 300 accessions) at four growth stages: seedling, bolting, flowering, and silique. This yielded a total of 400 plants (100 plants per stage). UAV imagery was acquired using a DJI Mavic 3T UAV (DJI, Shenzhen, China), which captured high-resolution videos at 30 frames per second (3840 × 2160 pixels). The UAV circled each plant three times at viewing angles of 0°, 30°, and 60° to ensure full surface coverage, as confirmed by preliminary tests for accurate 3D model reconstruction. The same approach was used to capture 3D models of the potted plants.

For field-level rapeseed populations, a UAV with a −60° viewing angle and a 5 m distance from the plot center was used for multi-view imaging. To ensure accurate 3D reconstruction, UAV image acquisition was conducted under calm conditions, with wind speeds below level 3 (3.4 m/s), to minimize canopy movement and support reliable multi-view image alignment. Images were captured every 10° along the flight path, resulting in 36 images per plot (8000 × 6000 pixels). Thirty-two plots were randomly selected from the 300 non-destructive sampling plots to evaluate similarity between simulated and real-world surface point clouds and to perform yield prediction based on silique-stage data. After UAV imaging, five field plots were destructively sampled. For each plot, the spatial positions of all plants were recorded, after which each individual plant was excavated, transplanted into pots, and reconstructed separately using our NeRF-based pipeline. These complete single-plant models were then merged according to their recorded spatial locations to generate a ground-truth population point cloud, which served as a benchmark for evaluating CP-PCN completion accuracy on real field data.

### Dataset generation for the rapeseed population point cloud completion model

To develop the rapeseed population point cloud completion model, we propose a VRI simulation method combined with an occlusion point detection algorithm to generate a comprehensive training dataset. Multi-view videos of individual rapeseed plants at four key growth stages (seedling, bolting, flowering, and silique) were captured using a UAV. Images were sampled at regular intervals to ensure full coverage of plant surfaces from different perspectives (Figure 1A). A structure-from-motion algorithm was used to estimate pose information for each image, which, together with the images, was input into the Instant-NGP model, a NeRF-based approach, to generate 3D models of individual plants, including point clouds and triangular mesh models (Jiang et al., 2020). To ensure that final point clouds preserve complete surface morphology, triangular meshes were first generated using the marching cubes algorithm, after which mesh vertices were extracted and uniformly sampled to create dense point clouds representing full surface geometry (Chen and Zhang, 2021). Representative mesh reconstruction results for the four growth stages are provided in Supplemental Figure 7.

The VRI method simulated real field plot configurations with 16 plants spaced 25 cm apart in rows separated by 28 cm. Plants from the individual dataset at the same growth stage were randomly selected, and the base of each plant bounding box—defined as the intersection of the main stem and root—was aligned with the simulated plot coordinates. When plants overlapped after placement, overlap was handled directly in the point domain by identifying spatially redundant points using a small distance threshold and retaining only one representative set of points in the overlapping region. Because point clouds are discrete samples in 3D space, this distance-based de-duplication provides a practical approximation of physical contact or attachment between neighboring structures, while preventing artificial point duplication and density inflation. The resulting merged point cloud therefore preserves realistic canopy overlap and maintains a consistent point distribution for subsequent dataset annotation and model training. This method generated diverse rapeseed population canopy architectures (Figure 1B). To create a point cloud completion dataset, we developed an occlusion point detection algorithm based on ray tracing. This algorithm identified surface points (visible from at least one camera perspective) and occluded points (completely invisible due to inter-plant occlusion). Ray–point intersections were calculated between each point and simulated camera viewpoints, which were generated based on the real UAV flight configuration described in the Experimental design and data acquisition section, ensuring that visibility annotation corresponds to actual multi-view imaging conditions. Surface points were used as input data, and occluded points were used as the model target output for completion (Figure 1C). Using the VRI simulation and occlusion point detection algorithm (Box 1), 4000 rapeseed population samples were generated (1000 per growth stage). Data from all growth stages were pooled and randomly divided into 80% for training and 20% for validation, enabling the completion network to learn generalizable canopy completion features across developmental stages. For five destructively sampled field plots, the individual plant videos were processed using the same 3D reconstruction pipeline to generate complete canopy models. These models were assembled using the VRI method, using recorded relative positions of each plant and the ground-truth complete point clouds for these plots were obtained.

Box 1 **Occlusion point detection**

**Input:** point cloud and triangle mesh of plant canopy, imaging coordinates.

**Output:** surface points, occluded points.

1: for each point in the plant point cloud:

2: for each imaging point:

3: for each triangle in the mesh model:

4: if a ray from the plant point to the imaging point intersects with the triangle:

5: Increment intersection count by 1;

6: end if

7: end for

8: if intersection count > 0:

9: Increment occluded direction by 1;

10: end if

11: End for

12: if occluded direction > total imaging points:

13: Mark the point as completely occluded and unavailable from multi-view 3D reconstruction;

14: end if

15: end for

### Evaluation of similarity between simulated and real point clouds

The SSIM3D was used to assess similarity between surface point clouds from UAV multi-view images and simulated population point clouds (Alexiou and Ebrahimi, 2020). The calculation formula is as follows:

(Equation 1) $$SSIM3D\left(X,Y\right)=\frac{1}{N}\sum_{P=1}^{N}\frac{\left|F_{X}\left(q\right)-F_{Y}\left(p\right)\right|}{\max\left(\left|F_{X}\left(q\right)\right|,\left|F_{Y}\left(p\right)\right|\right)+\varepsilon }$$

where *X* and *Y* represent two sets of 3D point clouds, *F*_*X*_(*q*) and *F*_*Y*_(*p*) denote feature values of points *q* and *p*, respectively, and *N* is the number of points in the point cloud. The term *ε* is a small value used to prevent division by zero.

For each of the 32 real field plots, SSIM3D values were calculated by comparing UAV-derived surface point clouds with 1000 simulated plots. The highest similarity score from these comparisons was retained as the final evaluation metric (Supplemental Figure 8). To ensure meaningful comparison, simulated point clouds were first processed using the occlusion point detection algorithm, and only visible surface point clouds were included in the comparison. This process ensured that the comparison focused on observable canopy regions, thereby reflecting the accuracy of simulated canopy architecture relative to real-world field data. The overall similarity score for each simulation approach was computed by averaging the highest SSIM3D values across all 32 plots. In addition to the VRI simulation, two conventional methods based on individual plant replication were also evaluated, one using potted plants and the other using field-grown plants. Given morphological differences between these plant types and variation across growth stages, comparisons were performed separately for each stage, enabling a detailed evaluation of each method’s performance in replicating realistic canopy architectures across developmental stages.

### Network architecture

To address highly variable and complex structural characteristics of rapeseed population canopy architectures across growth stages, we designed a deep learning network called CP-PCN, which integrates advantages of GANs and graph neural networks (Figure 2B). The CP-PCN architecture consists of two primary components, a generator and a discriminator. The generator predicted missing portions of the point cloud from the incomplete input, whereas the discriminator evaluated the plausibility of the generated point cloud by distinguishing it from the ground-truth data. To preserve plant architecture during down-sampling, a sliding window approach was used to segment large population point clouds into smaller, manageable blocks before processing (Figure 2A). This strategy maintained structural integrity and supported effective learning of local and global canopy features.

The input to the network is a point cloud containing 8192 points, which is first down-sampled twice using iterative farthest point sampling (Charles et al., 2017). This strategy supports consistent feature encoding while maintaining a balance between computational efficiency and model accuracy. This down-sampling yields two additional resolutions containing 4096 and 2048 points, capturing fine and global canopy features at different scales. After down-sampling, multi-resolution feature extraction is performed by an MRDG. The MRDG uses a DGCFE to learn local and global spatial relationships of points across the three resolutions (Wang et al., 2019). This enables the model to adaptively update neighborhood relationships and capture fine-grained geometric features at multiple scales (Figure 2C). Feature vectors from each resolution are then concatenated to form a 5760D latent vector *V*. This feature vector provides a compact representation of canopy architecture, enabling the network to handle the complexity and variability of plant architectures across growth stages.

The PPD is then used to decode this feature vector into three point clouds at three resolutions: coarse, middle, and fine. The coarse point cloud *Y*_*coarse*_ contains the primary center points, while the middle and fine point clouds *Y*_*middle*_ and *Y*_*fine*_ represent more detailed, refined architectures. The predicted point clouds at these resolutions have sizes of 2048, 4096, and 8192 points, respectively. These point clouds are progressively refined and up-sampled using fully connected layers in the PPD, ensuring that the final output closely resembles the ground truth, particularly in regions where canopy details were occluded. Finally, the completed point cloud is generated by merging the input point cloud with the predicted point cloud at a fine resolution. For more detailed information about the network architecture, including MRDG and PPD, please refer to Supplemental Note 8.

### Network training, validation, and testing

To train the CP-PCN model for point cloud completion, an effective loss function was designed to optimize both feature extraction and generation processes. The total loss function consisted of two components: a multi-stage completion loss and an adversarial loss. The completion loss was based on CD, a widely used metric for point cloud similarity, and was computed between predicted point clouds and the ground truth at three hierarchical resolutions (Wu et al., 2021). The formula for CD is

(Equation 2) $$d_{CD}\left(S_{1},S_{2}\right)=\frac{1}{S_{1}}\sum_{x\in S_{1}}\min_{y\in S_{2}}{\left\|x-y\right\|}_{2}^{2}+\frac{1}{S_{2}}\sum_{y\in S_{2}}\min_{x\in S_{1}}{\left\|y-x\right\|}_{2}^{2}\text{,}$$

where *S*_1_ and *S*_2_ represented two sets of 3D point clouds. Accordingly, the multi-stage completion loss was defined as

(Equation 3) $$L_{com}=d_{CD1}\left(Y_{fine},Y_{GT}\right)+\alpha \;d_{CD2}\left(Y_{middle},Y_{GT}^{\prime }\right)+2\alpha \;d_{CD3}(Y_{coarse},Y_{GT}^{\prime \prime }),$$

where *Y*_*fine*_, *Y*_*middle*_, and *Y*_*coarse*_ are the predicted point clouds at three resolutions and *Y*_*GT*_, $Y_{GT}^{\prime }$, and $Y_{GT}^{\prime \prime }$ are the corresponding ground truths obtained by sub-sampling using iterative farthest point sampling. The hyperparameter *α* was adjusted during training to balance contributions from different resolutions.

To further enhance realism of predicted architectures, an adversarial loss based on a GAN framework was incorporated. The generator *F*() aimed to reconstruct the missing region, while the discriminator *D*() attempted to distinguish the generated output from real samples. The adversarial loss was formulated as

(Equation 4) $$L_{adv}=\sum_{1\leq i\leq S}\log\left(D\left(y_{i}\right)\right)+\sum_{1\leq i\leq S}\log\left(1-D\left(F\left(x_{i}\right)\right)\right)\text{,}$$

where *x*_*i*_ and *y*_*i*_ represent input and ground-truth samples, respectively, and *S* is the batch size. The total loss function for model training was

(Equation 5) $$L=\lambda_{com}L_{com}+\lambda_{adv}L_{adv},$$

with *λ*_*com*_ = 0.9 and *λ*_*adv*_ = 0.1, ensuring a stronger emphasis on structural accuracy during training. For a more detailed loss function design process, please refer to Supplemental Note 9.

The CP-PCN network was implemented using PyTorch and trained on a workstation equipped with an Intel i9-10900K CPU and an NVIDIA RTX 3090 GPU. The Adam optimizer was used with an initial learning rate of 0.0001 and a batch size of 8. The model was trained on the simulated rapeseed population dataset, consisting of annotated surface and occluded point clouds. All network weights were randomly initialized due to the absence of publicly available pre-trained models for this task. Training was conducted for up to 200 epochs or until the loss dropped below 0.1.

During training, batch normalization and rectified linear unit (ReLU) activation were used in the MRDG and discriminator, while the PPD used only ReLU activations except at the output layer. The weighting parameter *α* for the multi-resolution loss was dynamically adjusted: 0.01 for epochs 0–30, 0.05 for epochs 30–80, and 0.1 for epochs beyond 80. The validation set was evaluated every 10 epochs; if the loss decreased, model parameters were updated to reduce overfitting and support generalization.

Once trained, the CP-PCN model was applied to surface point clouds of five destructively sampled rapeseed plots reconstructed from UAV imagery. The model completed occluded regions, and predicted points were merged with the input to form complete 3D reconstructions. These predictions were then quantitatively compared with the ground truth to evaluate real field performance (Supplemental Figure 9).

### Ablation study and hyperparameter analysis

To evaluate the contribution of each component in the proposed CP-PCN architecture, a comprehensive ablation study was conducted. Specifically, four variants of the model were designed. First, to assess the effect of multi-resolution encoding, MRDG was replaced with a single-resolution encoder. Second, to examine the role of DGCFE, it was substituted with a standard MLP block for local feature extraction. Third, to test the impact of PPD, it was replaced with a single fully connected layer for coarse-to-fine reconstruction. Finally, to assess the influence of the GAN-based loss, the discriminator was removed, and the model was trained using only reconstruction loss functions. All variants were trained and tested under identical experimental settings, and completion performance was compared in terms of CD.

In addition, we conducted a sensitivity analysis on four hyperparameters: (i) the number of nearest neighbors (*k*) in dynamic graph convolution, tested with values {8, 10, 12, 16, 20, 24, 28, 32}; (ii) the input (*N*) and output (*M*) point cloud sizes, varied across {2048, 4096, 8192, 16384}; and (iii) the number of layers (*O*) in the MRDG, set to {4, 5, 6, 7}. Based on prior research, the first five layers were set to {64, 128, 256, 512, 1024}. For deeper configurations with six and seven layers, additional layers were set to 2048 and 4096, respectively, to increase feature extraction capacity. Each parameter was varied independently while keeping the others constant to isolate its effect on model performance.

### Comparison analysis

To further evaluate the effectiveness of CP-PCN, we benchmarked it against several state-of-the-art point cloud completion models, including PF-Net, GRNet, and PoinTr (Huang et al., 2020; Xie et al., 2020; Yu et al., 2021). These models were selected because they represent distinct paradigms in point cloud completion. PF-Net, a lightweight model, uses a GAN-based architecture with MLP-based feature extraction to generate missing regions. GRNet employs a deep residual network structure, inspired by ResNet, to enhance precision through hierarchical feature learning. In contrast, PoinTr incorporates a transformer-based architecture, achieving high accuracy but at the cost of increased computational complexity. All models were trained and tested under identical conditions, ensuring a fair comparison. Performance was evaluated based on visual inspection, CD, and computational efficiency, allowing a comprehensive assessment of reconstruction quality and model efficiency.

### CP-PCN for yield estimation

To evaluate whether CP-PCN improves agronomic trait extraction, we examined the accuracy of yield estimationusing completed canopy point clouds in 32 non-destructively sampled field plots. Because silique architecture is the dominant contributor to rapeseed yield, SEI was proposed as a structural proxy for yield potential. SEI is defined as total silique volume per unit ground area of the plot, with the following formula:

(Equation 6) siliqueefficiencyindex=siliquevolume/planearea.

Silique voxels were extracted from population point clouds using a previously developed organ segmentation model (Du et al., 2023). Silique volume was calculated using voxel-based solid volume estimation. To ensure fair comparison among incomplete, PoinTr-completed, and CP-PCN-completed point clouds, a fixed voxel size of 3 mm, as suggested by reported studies, was used for all samples after metric calibration. This resolution is approximately half of the typical silique diameter, allowing voxelization to represent occupied silique space while minimizing artificial hollowing caused by missing points.

After harvest, siliques from each plot were manually collected, dried, and weighed to obtain ground-truth yield. SEI values derived from incomplete, PoinTr-based, and CP-PCN-based completed point clouds were regressed against measured yield, and the coefficient of determination (R^2^) was used as the evaluation metric.

## Data and code availability

All source code and test data used in this study are publicly available on GitHub (https://github.com/Ziyue-Guo/CP-PCN.git).

## Funding

This work was supported by the 10.13039/501100001809National Natural Science Foundation of China (Grant No. 32371985) and the International S&T Cooperation Program of China (Grant No. 2024YFE0115000).

## Acknowledgments

We extend our heartfelt gratitude to Ruisen Wang, Yi Feng, Mengjie Gong, and Guangyu Wu for their participation in the experiments, and to the Jiaxing Academy of Agricultural Sciences for assistance with experimental data acquisition. This work was supported by the Earth System Big Data Platform of the School of Earth Sciences, Zhejiang University. The authors declare that they have no known competing financial interests or personal relationships that could have appeared to influence the work reported in this paper.

## Author contributions

Methodology, Z.G.; software, Z.G. and X.Y.; validation, Z.G. and X.Y.; formal analysis, Z.G.; investigation, Z.G., X.Y., and Y.S.; data curation, Z.G. and X.Y.; writing – original draft, Z.G.; writing – review & editing, H.C.; visualization, Z.G.; resources, Y.Z. and L.J.; conceptualization, H.C.; supervision, H.C.; project administration, H.C.; funding acquisition, H.C.

## Supplemental information

Document S1. Supplemental Figures 1–9 and Supplemental Notes 1–9

Document S2. Article plus supplemental information

## References

[bib1] Alexiou E., Ebrahimi T. (2020). 2020 IEEE International Conference on Multimedia & Expo Workshops (ICMEW).

[bib2] Arshad M.A., Jubery T., Afful J., Jignasu A., Balu A., Ganapathysubramanian B., Sarkar S., Krishnamurthy A. (2024). Evaluating Neural Radiance Fields for 3D Plant Geometry Reconstruction in Field Conditions. Plant Phenom..

[bib3] Bailey B.N., Mahaffee W.F. (2017). Rapid measurement of the three-dimensional distribution of leaf orientation and the leaf angle probability density function using terrestrial LiDAR scanning. Rem. Sens. Environ..

[bib4] Charles R.Q., Su H., Kaichun M., Guibas L.J. (2017). 2017 IEEE Conference on Computer Vision and Pattern Recognition (CVPR).

[bib5] Chen Z., Zhang H. (2021). Neural marching cubes. ACM Trans. Graph..

[bib6] Chen H., Liu S., Wang C., Wang C., Gong K., Li Y., Lan Y. (2023). Point Cloud Completion of Plant Leaves under Occlusion Conditions Based on Deep Learning. Plant Phenomics.

[bib7] Du R., Ma Z., Xie P., He Y., Cen H. (2023). PST: Plant segmentation transformer for 3D point clouds of rapeseed plants at the podding stage. ISPRS J. Photogrammetry Remote Sens..

[bib8] Fei B., Yang W., Chen W.-M., Li Z., Li Y., Ma T., Hu X., Ma L. (2022). Comprehensive Review of Deep Learning-Based 3D Point Cloud Completion Processing and Analysis. IEEE trans. Intell. Transp. Syst..

[bib9] Ge Y., Xiong Y., From P.J. (2020). Symmetry-based 3D shape completion for fruit localisation for harvesting robots. Biosyst. Eng..

[bib10] Geiger A., Lenz P., Stiller C., Urtasun R. (2013). Vision meets robotics: The KITTI dataset. Int. J. Robot Res..

[bib11] Huang Z., Yu Y., Xu J., Ni F., Le X. (2020). 2020 IEEE/CVF Conference on Computer Vision and Pattern Recognition (CVPR).

[bib12] Jiang S., Jiang C., Jiang W. (2020). Efficient structure from motion for large-scale UAV images: A review and a comparison of SfM tools. ISPRS J. Photogrammetry Remote Sens..

[bib13] Jin S., Sun X., Wu F., Su Y., Li Y., Song S., Xu K., Ma Q., Baret F., Jiang D. (2021). Lidar sheds new light on plant phenomics for plant breeding and management: Recent advances and future prospects. ISPRS J. Photogrammetry Remote Sens..

[bib14] Li M., Shamshiri R.R., Schirrmann M., Weltzien C., Shafian S., Laursen M.S. (2022). UAV Oblique Imagery with an Adaptive Micro-Terrain Model for Estimation of Leaf Area Index and Height of Maize Canopy from 3D Point Clouds. Remote Sens..

[bib15] Li N., Li J., Tung S.A., Shi X., Hao X., Shi F., Wahid M.A., Ali B., Rashid R., Wang J., Luo H. (2022). Optimal irrigation amount can increase cotton lint yield by improving canopy structure and microenvironment under non-film deep drip irrigation. J. Clean. Prod..

[bib16] Li L., Liu G., Xu F., Deng L. (2024). CarvingNet: Point cloud completion by stepwise refining multi-resolution features. Pattern Recogn..

[bib17] Lin G., Li H., Yang Z., Ruan Y., Liu C. (2024). Pod canopy staggered-layer cultivation increases rapeseed (*Brassica napus* L.) yield by improving population canopy structure and fully utilizing light-energy resources. Eur. J. Agron..

[bib18] Liu F., Song Q., Zhao J., Mao L., Bu H., Hu Y., Zhu X.-G. (2021). Canopy occupation volume as an indicator of canopy photosynthetic capacity. New Phytol..

[bib19] Long X., Liu L., Theobalt C., Wang W., Vedaldi A., Bischof H., Brox T., Frahm J.-M. (2020). Computer Vision – ECCV 2020.

[bib20] Lou M., Lu J., Wang L., Jiang H., Zhou M. (2022). Growth parameter acquisition and geometric point cloud completion of lettuce. Front. Plant Sci..

[bib21] Ma Z., Du R., Xie J., Sun D., Fang H., Jiang L., Cen H. (2023). Phenotyping of Silique Morphology in Oilseed Rape Using Skeletonization with Hierarchical Segmentation. Plant Phenomics.

[bib22] Macedo M.C.F., Apolinário A.L. (2023). Occlusion Handling in Augmented Reality: Past, Present and Future. IEEE Trans. Vis. Comput. Graph..

[bib23] Magistri F., Marcuzzi R., Marks E., Sodano M., Behley J., Stachniss C. (2024). 2024 IEEE International Conference on Robotics and Automation (ICRA).

[bib24] Masuda T. (2021).

[bib25] Murchie E.H., Burgess A.J. (2022). Casting light on the architecture of crop yield. Crop Environ..

[bib26] Paullada A., Raji I.D., Bender E.M., Denton E., Hanna A. (2021). Data and its (dis)contents: A survey of dataset development and use in machine learning research. Patter.

[bib27] Rossi R., Costafreda-Aumedes S., Summerer S., Moriondo M., Leolini L., Cellini F., Bindi M., Petrozza A. (2022). A comparison of high-throughput imaging methods for quantifying plant growth traits and estimating above-ground biomass accumulation. Eur. J. Agron..

[bib28] Sarmad M., Lee H.J., Kim Y.M. (2019). RL-GAN-Net: A Reinforcement Learning Agent Controlled GAN Network for Real-Time Point Cloud Shape Completion. arXiv.

[bib29] Sun B., Wang C., Yang C., Xu B., Zhou G., Li X., Xie J., Xu S., Liu B., Xie T. (2021). Retrieval of rapeseed leaf area index using the PROSAIL model with canopy coverage derived from UAV images as a correction parameter. Int. J. Appl. Earth Obs. Geoinf..

[bib30] Tchapmi L.P., Kosaraju V., Rezatofighi H., Reid I., Savarese S. (2019).

[bib31] Wang Y., Sun Y., Liu Z., Sarma S.E., Bronstein M.M., Solomon J.M. (2019). Dynamic Graph CNN for Learning on Point Clouds. ACM Trans. Graph..

[bib32] Wang C., Xu S., Yang C., You Y., Zhang J., Kuai J., Xie J., Zuo Q., Yan M., Du H. (2024). Determining rapeseed lodging angles and types for lodging phenotyping using morphological traits derived from UAV images. Eur. J. Agron..

[bib33] Watawana B., Isaksson M. (2024). Automated microgreen phenotyping for yield estimation using a consumer-grade depth camera. Smart Agric. Technol..

[bib34] Wu Z., Song S., Khosla A., Yu F., Zhang L., Tang X., Xiao J. (2015). 3D ShapeNets: A Deep Representation for Volumetric Shapes. arXiv.

[bib35] Wu T., Pan L., Zhang J., WANG T., Liu Z., Lin D. (2021). Advances in Neural Information Processing Systems.

[bib36] Xie H., Yao H., Zhou S., Mao J., Zhang S., Sun W., Vedaldi A., Bischof H., Brox T., Frahm J.-M. (2020). Computer Vision – ECCV 2020.

[bib37] Yu X., Rao Y., Wang Z., Liu Z., Lu J., Zhou J. (2021). 2021 IEEE/CVF International Conference on Computer Vision (ICCV).

[bib38] Zhang Y., Huang D., Wang Y. (2021). PC-RGNN: Point Cloud Completion and Graph Neural Network for 3D Object Detection. Proc. AAAI Conf. Artif. Intell..

[bib39] Zhang Y., Su W., Tao W., Li Z., Huang X., Zhang Z., Xiong C. (2023). Completing 3D Point Clouds of Thin Corn Leaves for Phenotyping Using 3D Gridding Convolutional Neural Networks. Remote Sens..

[bib40] Zheng M., Terzaghi W., Wang H., Hua W. (2022). Integrated strategies for increasing rapeseed yield. Trends Plant Sci..

