# Supplementary material for: A novel point cloud completion model for three-dimensional reconstruction of complex, dynamic population-level crop canopy architecture
Source: Plant Commun. 2025 Dec 11;7(3):101675. doi: 10.1016/j.xplc.2025.101675 (PMC12983250; doi:10.1016/j.xplc.2025.101675)
Supplement: Document S1. Supplemental Figures 1–9 and Supplemental Notes 1–9 [file mmc1.pdf]

Plant Communications, Volume 7

Supplemental information

A novel point cloud completion model for three-dimensional reconstruction of complex, dynamic population-level crop canopy architecture

Ziyue Guo, Xin Yang, Yutao Shen, Yang Zhu, Lixi Jiang, and Haiyan Cen

Supplemental Figure

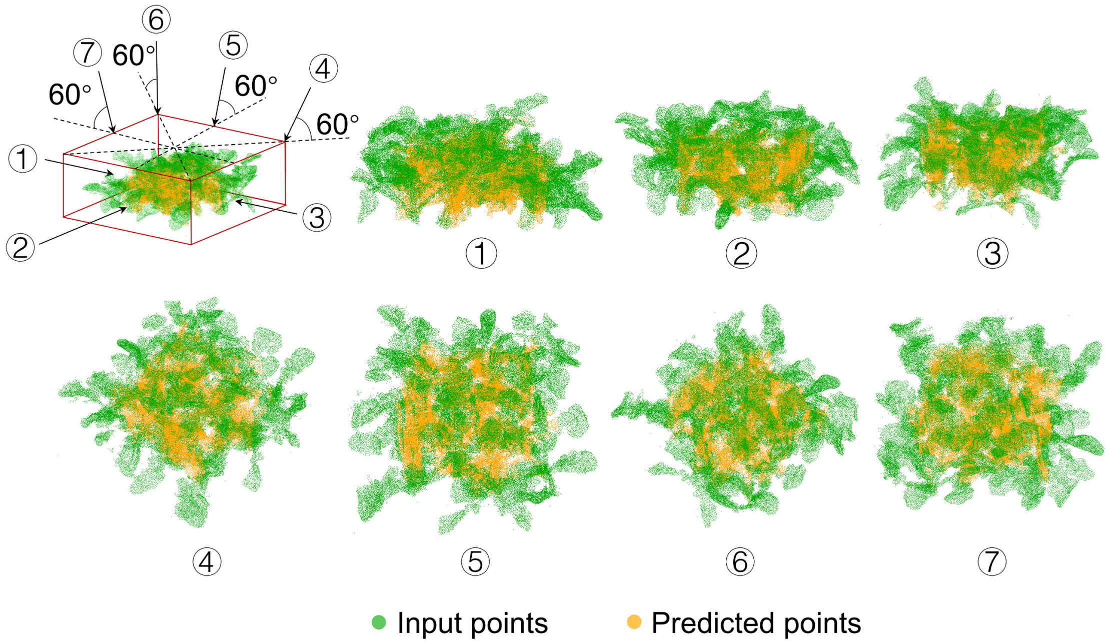

Supplemental Figure 1. Completion results of rapeseed populations at the seedling stage from multiple viewing angles.

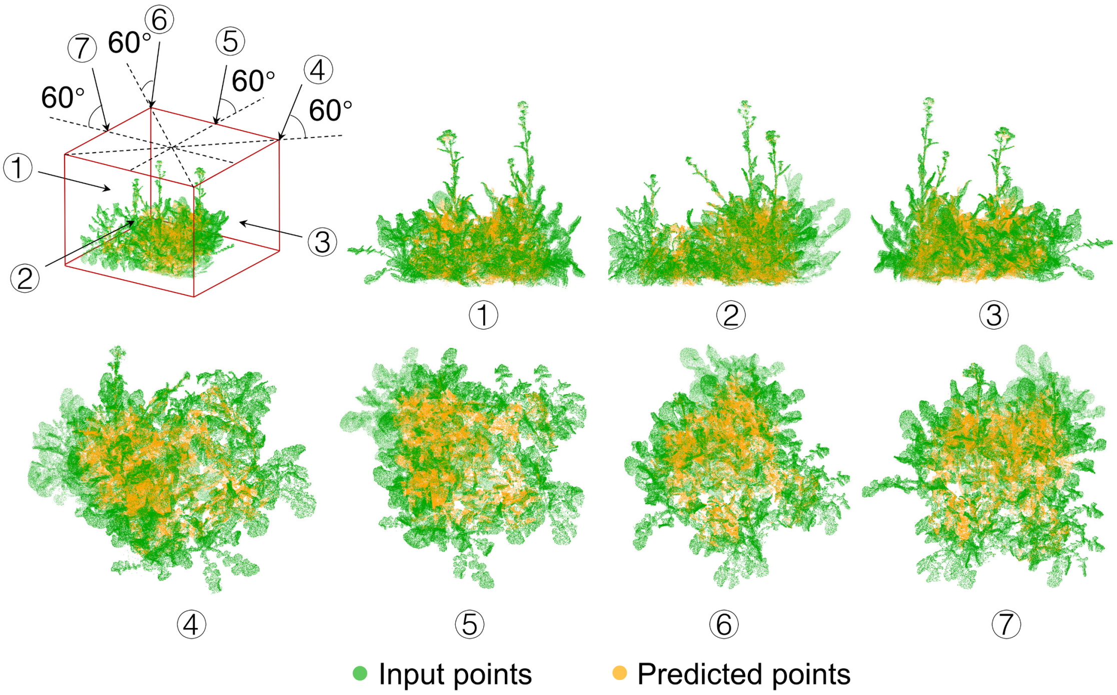

Supplemental Figure 2. Completion results of rapeseed populations at the bolting stage from multiple viewing angles.

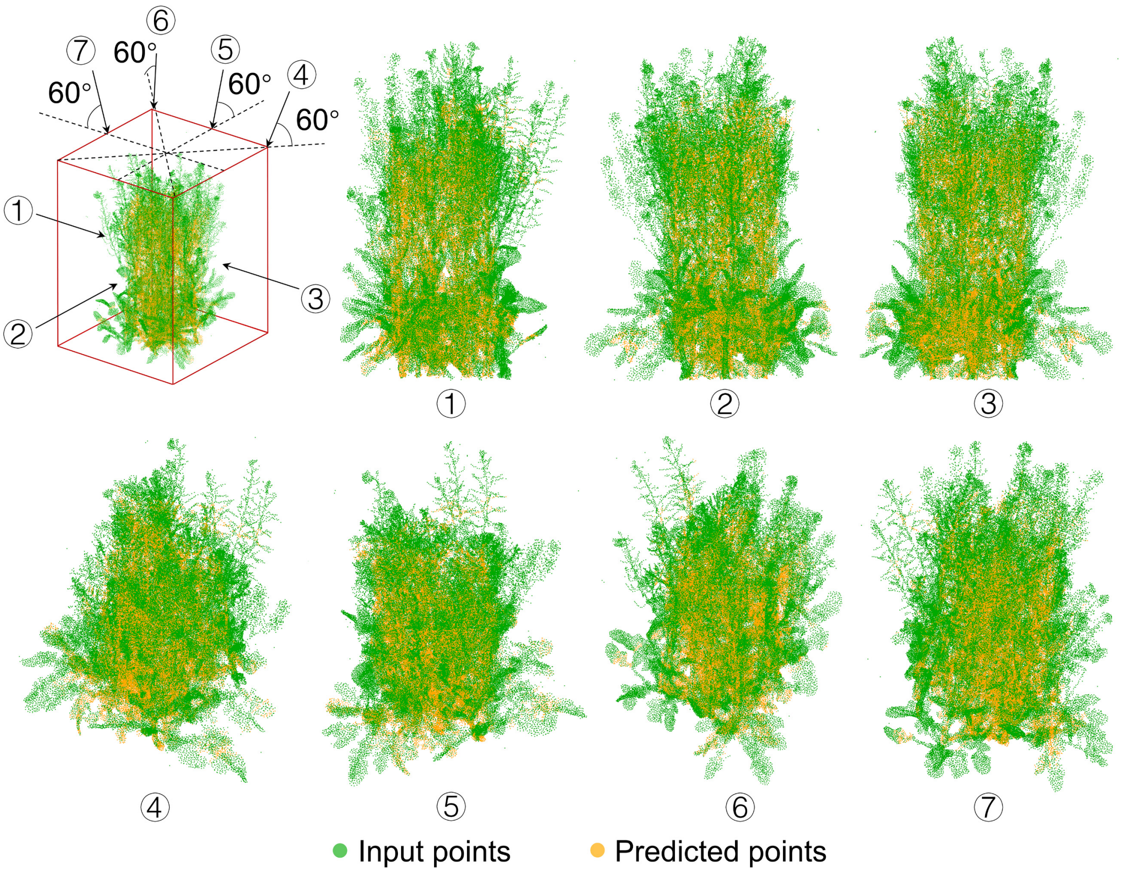

Supplemental Figure 3. Completion results of rapeseed populations at the flowering stage from multiple viewing angles.

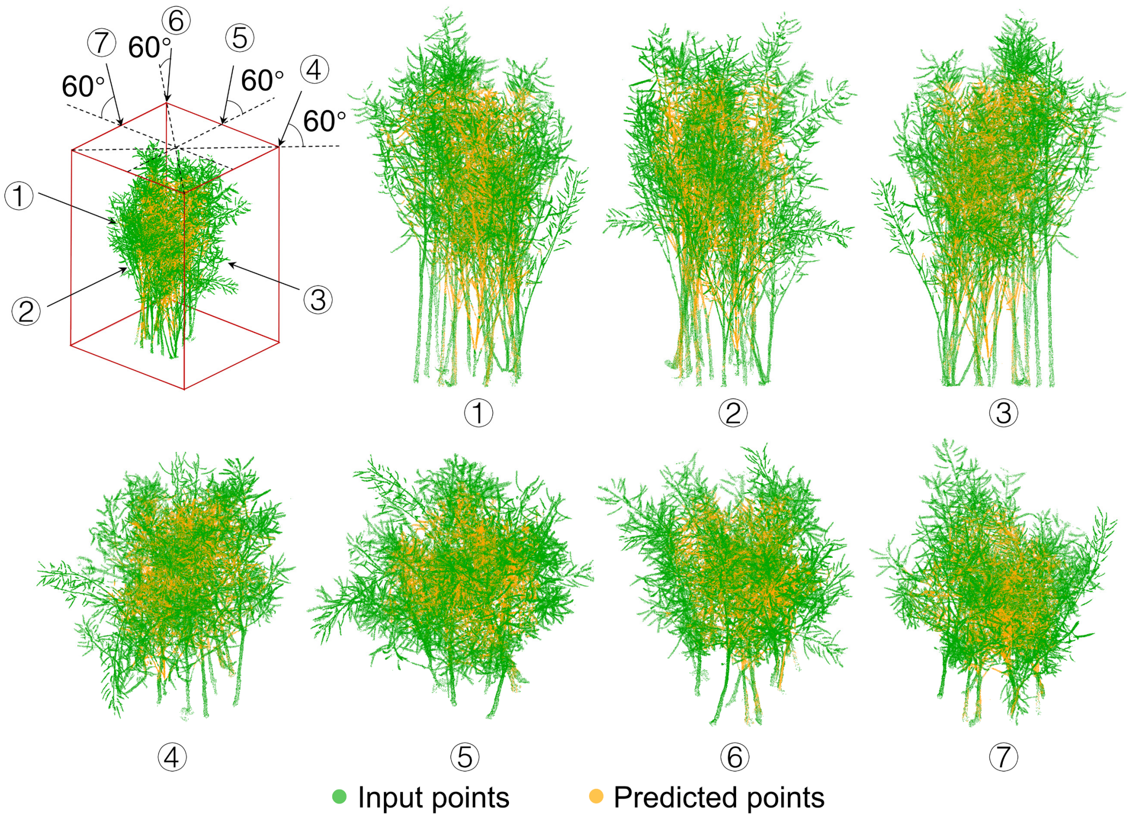

Supplemental Figure 4. Completion results of rapeseed populations at the silique stage from multiple viewing angles.

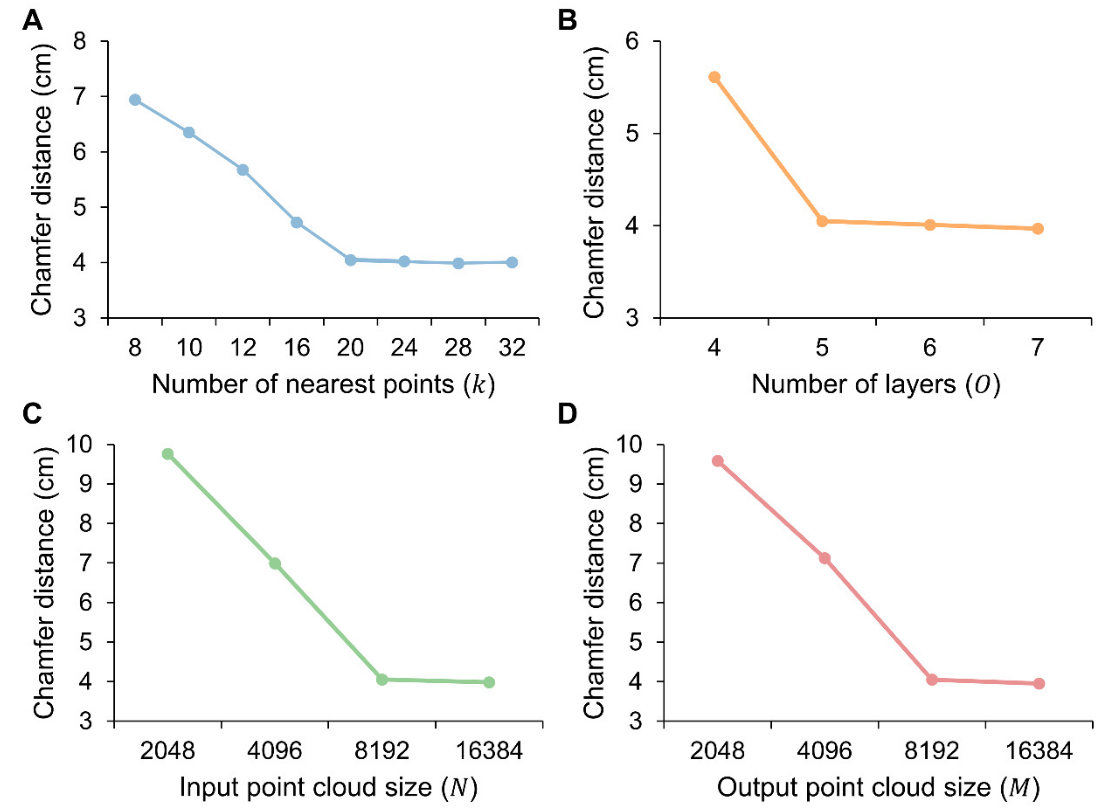

Supplemental Figure 5. Sensitivity analysis of (a) the number of nearest neighbors (k), (b) number of layers in the encoder (O), (c) input point cloud size (N), and (d) output point cloud size (M) on the model performance.

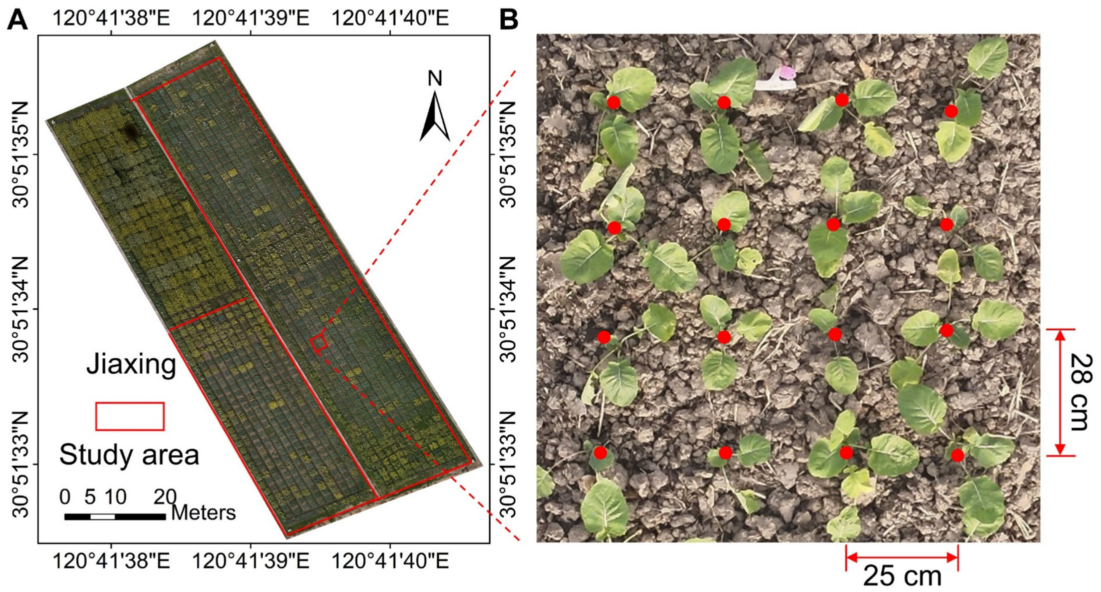

Supplemental Figure 6. Location of the experimental field (**A**) in Jiaxing, Zhejiang province, China with (**B**) the rapeseed plant layout within a plot. Each plot contains 16 rapeseed plants with the spacing of 28×25 cm.

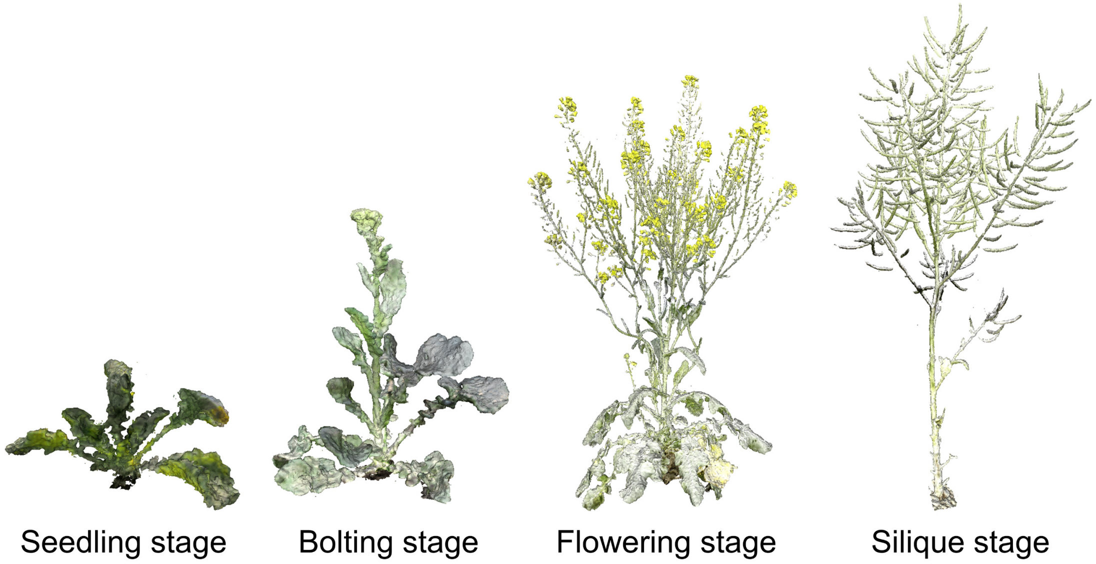

Supplemental Figure 7. Mesh models of rapeseed plants reconstructed using the marching cubes algorithm across four growth stages.

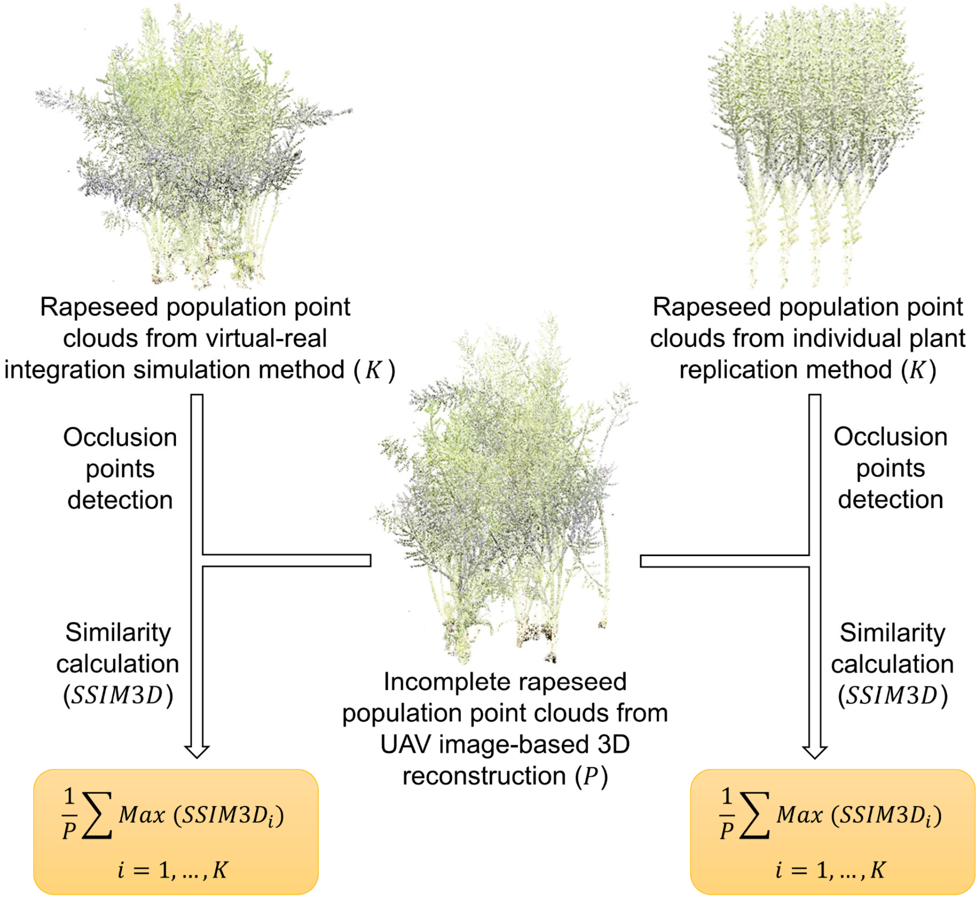

Supplemental Figure 8. Quantitative evaluation of simulated rapeseed population point clouds by comparing them with UAV-derived ground-truth using the three-dimensional structural similarity index (SSIM3D). The proposed virtual-real integration (VRI) simulation method was compared against traditional individual plant replication approaches.

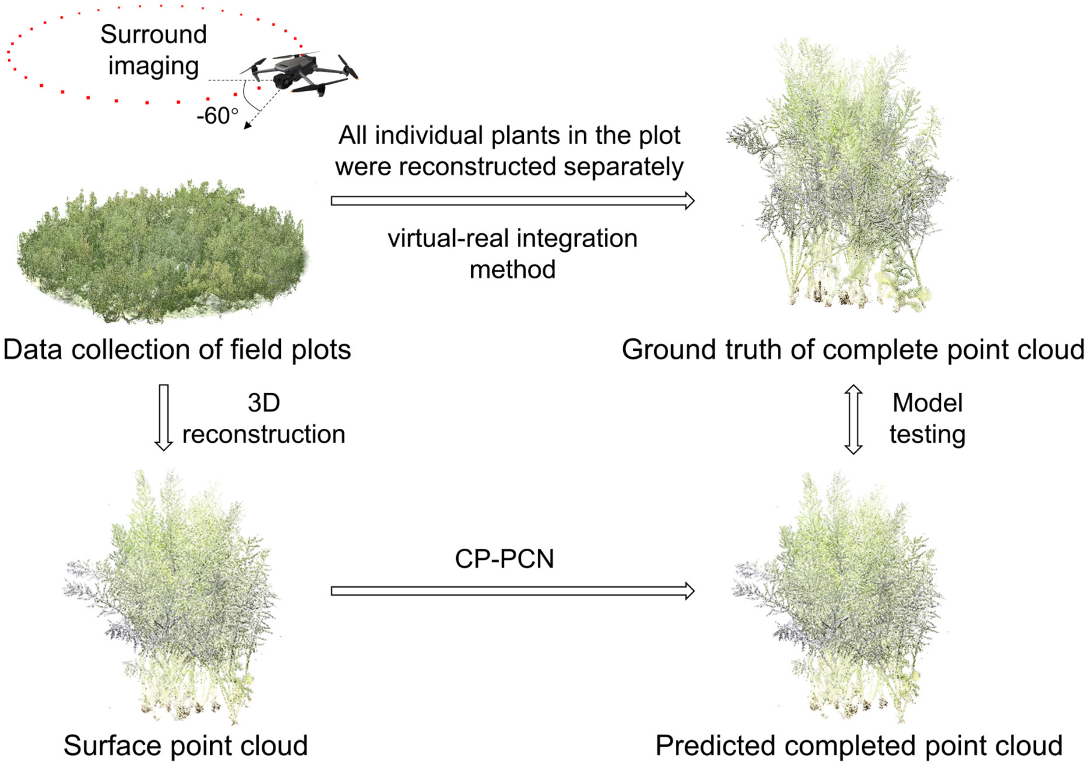

Supplemental Figure 9. Model testing pipeline of the crop population point cloud completion network (CP-PCN) using field plot data.

Supplemental Note

Supplemental Note 1. Visualization of CP-PCN completion results

Figure N1 presents the point cloud completion results generated by CP-PCN at different growth stages. Through slicing along the X, Y, and Z planes, the internal completion of the crop population canopy is showcased. It is evident that CP-PCN performs well in reconstructing both relatively simple architectures during the seedling stage and the more complex architectures during the flowering and silique stages. This highlights the model's robust capability to effectively handle a wide range of canopy complexities.

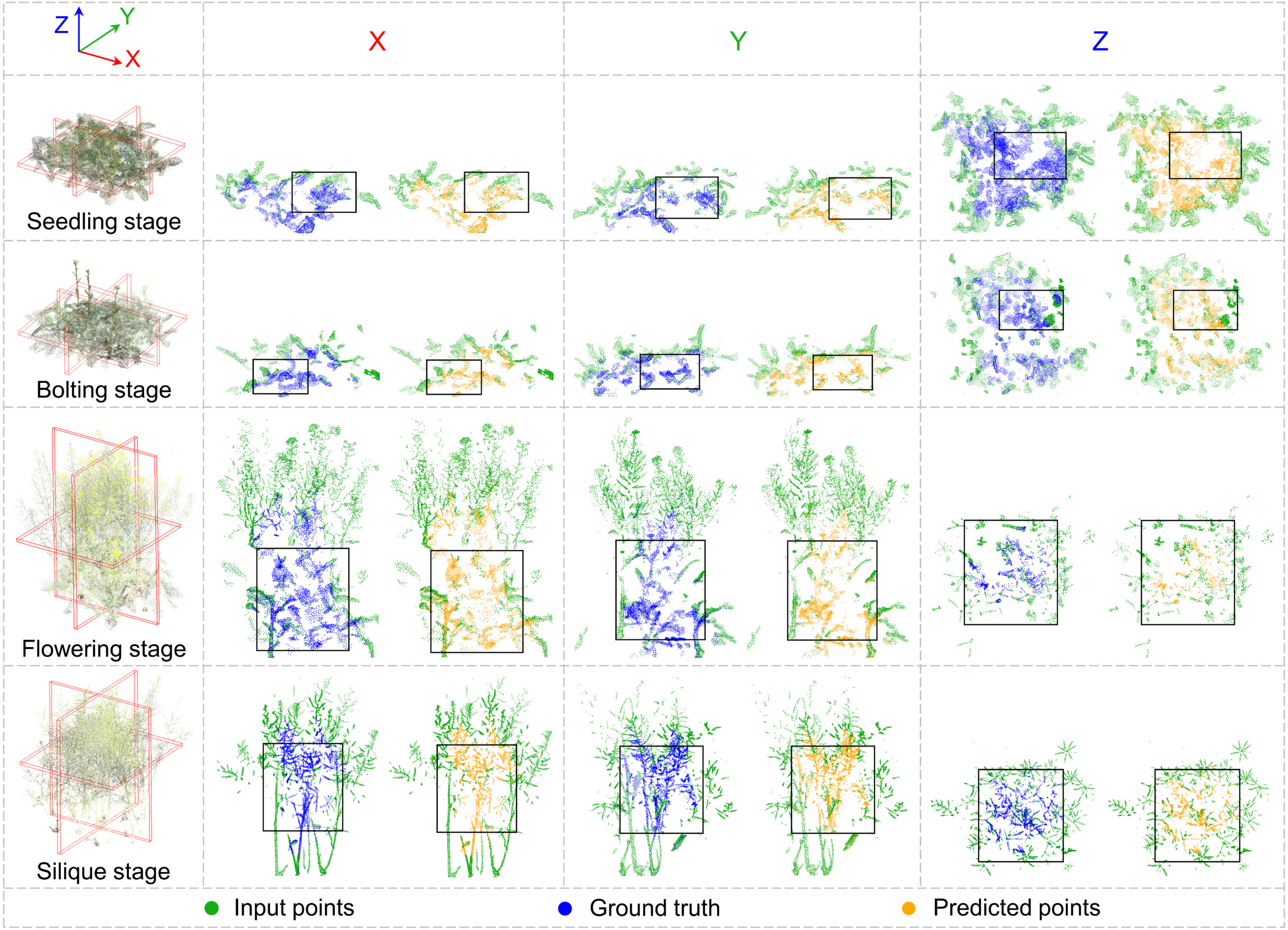

Z
Y
X
X
Y
Z
Seedling stage
Bolting stage
Flowering stage
Silique stage
Input points
Ground truth
Predicted points

Figure N1. Visualization of CP-PCN point cloud completion results across different growth stages. The input points (green), ground truth (blue), and predicted points (orange) are shown for each stage: seedling, bolting, flowering, and silique. The black boxes highlight the magnified regions displayed in Figure 3, where the detailed comparisons between ground truth and predicted points are further illustrated.

Supplemental Note 2. Effect of dataset generation strategy on model performance

To evaluate the influence of dataset generation strategy on the performance of CP-PCN, we prepared two training datasets based on the same VRI-generated complete population point clouds but with different missing-data constructions. In the random clipping dataset, incomplete point clouds were obtained by randomly removing approximately 45% of points from the complete canopies. In the visibility-based occlusion dataset, missing regions were generated by identifying canopy points that were unobservable from all simulated UAV viewpoints under the acquisition geometry, and only these occluded points were removed to form the incomplete inputs. Both models were trained using identical configurations and tested on the same benchmark dataset across four growth stages. Table N1 shows that the model trained with visibility-based occlusion labeling achieved much lower CD values across all stages (3.35 – 4.51 cm) than the model trained with random clipping (19.32 – 33.28 cm), demonstrating that realistic occlusion patterns and accurate labeling substantially improve training effectiveness and robustness under field-like conditions.

Table N1. Comparison of model performance under different dataset generation strategies based on chamfer distance (CD).

Dataset generation method	CD (cm)				
	Seedling stage	Bolting stage	Flowering stage	Silique stage	All
Random cropping	19.32	22.48	28.64	33.28	27.59

**Ours (occlusion-based)**	**3.35**	**3.46**	**4.32**	**4.51**	**4.05**

Supplemental Note 3. Ablation results of network components

The ablation results are summarized in Table N2. The full model integrating all four components, including MRDG, DGCFE, PPD, and GAN, achieved the best overall performance, with CD values ranging from 3.35 to 4.51 cm across the four growth stages. Removing any single component led to an obvious decline in completion accuracy. Among individual modules, DGCFE and MRDG contributed the largest single-module improvements, reducing mean CD by approximately 8–9 cm compared with the baseline configuration without any component. When modules were combined, the synergy between the feature extraction (MRDG, DGCFE) and decoding (PPD) components further enhanced reconstruction fidelity, lowering mean CD to 5.6–8.1 cm depending on the combination. Incorporating the GAN-based loss function provided additional gains in structural smoothness and global coherence, with the three-module combination of DGCFE+PPD+GAN yielding near-optimal results.

Table N2. Ablation results of CP-PCN under different module configurations based on chamfer distance (CD).

Modules				CD (cm)				
MRDG	DGCNN	PDD	GAN	Seedling stage	Bolting stage	Flowering stage	Silique stage	All
				15.82	16.34	18.56	19.24	17.52
√				9.86	9.94	10.48	10.73	10.37
	√			8.54	8.67	9.23	9.35	9.08
		√		10.12	10.33	10.87	11.06	10.67
			√	9.24	9.35	9.86	9.94	9.64
√	√			7.78	7.92	8.23	8.35	8.12
√		√		7.24	7.35	7.64	7.78	7.58
√			√	6.86	6.97	7.36	7.42	7.26

	√	√		6.38	6.45	6.67	6.82	6.67
	√		√	5.98	6.08	6.43	6.54	6.38
		√	√	5.33	5.42	6.04	6.21	5.85
√	√	√		5.22	5.34	5.85	5.94	5.63
√	√		√	5.18	5.36	5.74	5.89	5.54
√		√	√	5.01	5.01	5.79	5.89	5.36
	√	√	√	3.84	4.01	4.63	4.72	4.36
√	√	√	√	**3.35**	**3.46**	**4.32**	**4.51**	**4.05**

Note: MRDG, multi-resolution dynamic graph convolutional encoder; DGCFE, dynamic graph convolutional feature extractor; PPD, point pyramid Decoder; GAN, generative adversarial network–based loss.

Supplemental Note 4. Model performance under different planting densities

To examine the influence of canopy occlusion on model performance, point cloud datasets were generated under different planting densities by systematically varying row spacing and plant spacing. For each configuration, the average missing ratio of the input point cloud was quantified based on occlusion point detection algorithm, and CP-PCN was evaluated using CD. The resulting missing ratios ranged from 26% to 73%. The detailed quantitative results across planting densities are provided in Table N3. These data establish the relationship between planting geometry, missing ratio, and completion accuracy, and demonstrate the threshold selection noted in the main text.

Table N3. Effect of planting density on point cloud completion accuracy of CP-PCN (16 plants, 4×4 layout).

Row spacing (cm)	Plant spacing (cm)	Mean missing ratio (%)	CD (cm)
12	10	72.63	25.36
14	12	68.30	17.28
16	14	64.86	14.32
18	16	60.35	10.86
20	18	55.69	7.33
22	20	51.28	5.58

24	22	45.28	4.62
26	24	42.67	4.18
28	26	37.34	4.09
30	28	31.58	4.33
32	30	26.46	4.52

Note: CD, chamfer distance

Supplemental Note 5. Computational efficiency of the CP-PCN framework

The computational efficiency of the proposed CP-PCN pipeline was assessed to ensure its suitability for high-throughput phenotyping applications. As summarized in Supplementary Table N4, the complete workflow, including model construction and field application, was benchmarked on the experimental hardware used in this study. The model construction process, which includes single-plant image acquisition, pose estimation, NeRF training, dataset preparation, and network training, required approximately 15 hours in total. For model application at the plot level, UAV-based multi-view image acquisition and reconstruction required approximately 6 minutes, while point cloud completion inference took only 2 seconds. These results demonstrate that CP-PCN achieves a favorable trade-off between computational efficiency and reconstruction accuracy, making it well suited for large-scale, multi-plot canopy analysis.

Table N4. Processing time for model construction and application phases.

	Step	Time cost
Model Construction	Single-plant image acquisition	90 seconds / video
	Pose estimation	300 seconds / 150 images
	NeRF training	180 seconds
	Building point cloud completion data set	120 seconds / plot

	Training point cloud completion model	15 hours
Model Application	Plot-level image acquisition (UAV)	90 seconds / 36 images
	Pose estimation	100 seconds / 36 images
	NeRF training	180 seconds
	Model inference	2 seconds
	Completing a full breeding plot	20 seconds

Supplemental Note 6. Cross-species validation of CP-PCN using rice populations

To demonstrate that the proposed framework is applicable beyond rapeseed, we conducted a cross-species validation using rice (*Oryza sativa* L.), which has a canopy structure that is substantially different from rapeseed, including narrow upright leaves and dense panicle clusters at the maturity stage. Using the same dataset generation and occlusion annotation pipeline described in the main manuscript, a rice population point cloud completion dataset was generated. CP-PCN was retrained on the rice dataset. As shown in Figure N2A, CP-PCN successfully reconstructed canopy regions that were missing in the surface point clouds and preserved fine structures in both the panicle and stem areas. A representative individual plant extracted from the four viewing directions (front, back, left, and right) of the completed point cloud population is visualized in Figure N2B. it is observed that CP-PCN successfully reconstructed canopy regions that were occluded in the input point clouds.

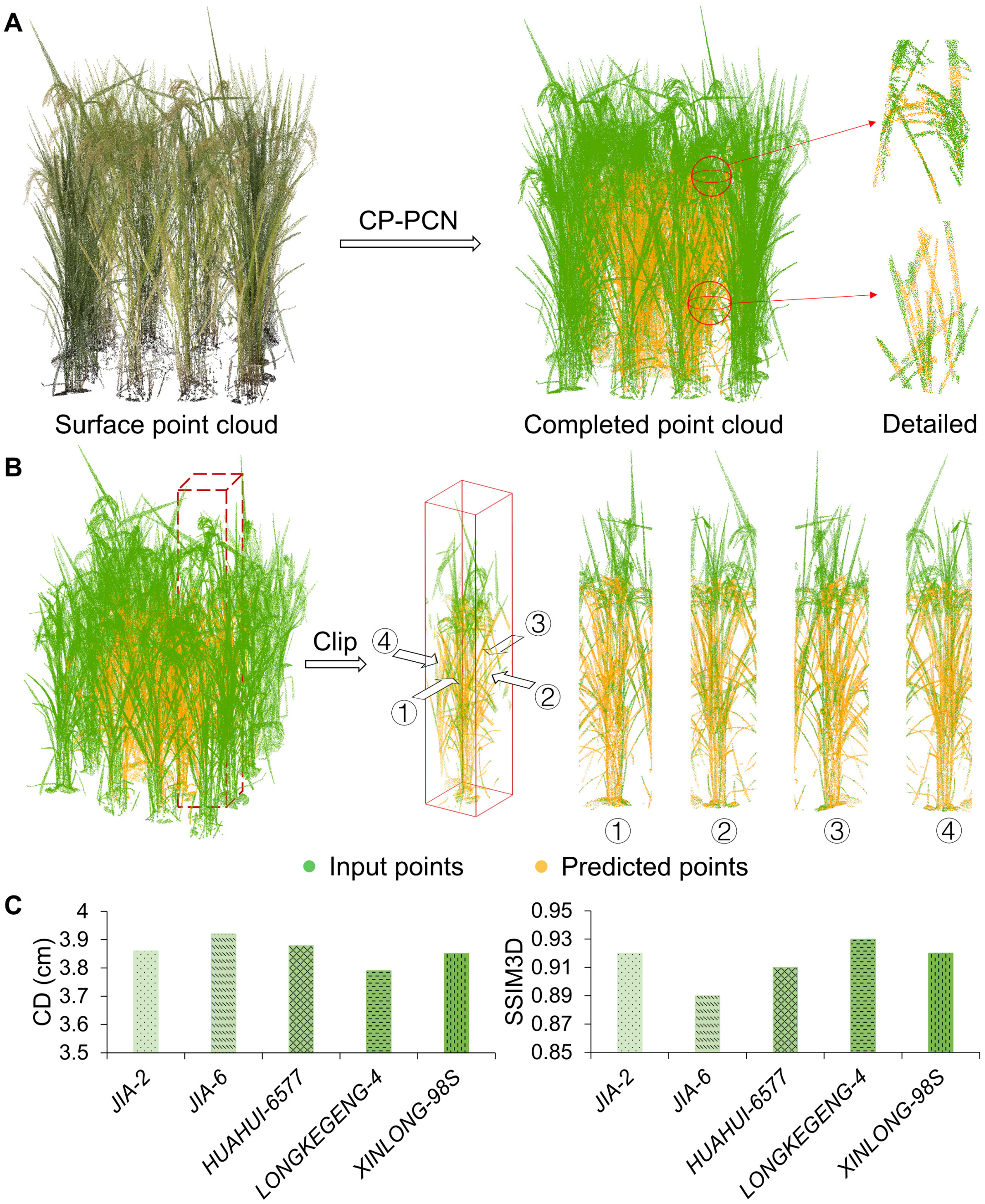

Figure N2. Cross-species validation of crop population point cloud completion network (CP-PCN) on rice populations. (**A**) Completed population canopy produced by CP-PCN retrained on a rice dataset generated with the same virtual–real integration (VRI) pipeline as rapeseed. (**B**) Representative rice plant cropped from the completed population and visualized from four viewing

directions (front, back, left, right). (**C**) Quantitative evaluation of completion accuracy across five rice cultivars using chamfer distance (CD) and three-dimensional structural similarity index (SSIM3D)

Quantitative evaluation was conducted using destructive sampling on five rice cultivars, and the results are summarized in Figure N2C. The completed point clouds achieved an average CD of 3.86 cm and a SSIM3D of 0.91. These values demonstrate strong spatial consistency between the completed point clouds and the ground-truth canopy structure.

Supplemental Note 7. Improvement of leaf area index (LAI) estimation using completed population point clouds

LAI estimation from completed population point clouds

To evaluate whether canopy completion improves physiological trait estimation, we calculated LAI using incomplete, PoinTr-based, and CP-PCN–based completed point clouds of rapeseed population.

Leaf points were segmented from plot-level canopy point clouds using a point-cloud semantic segmentation model (PST). Segmented leaf points were converted into triangular meshes via Poisson surface reconstruction, from which the total leaf surface area A_{leaf} was computed. LAI was then calculated as:

$$LAI = {A_{leaf}}/{A_{ground}}$$

where A_{ground} represents the horizontal area of the breeding plot. LAI validation was performed using the same 32 destructively sampled plots as in

the yield experiment. Ground-truth LAI was measured using an LAI-2200C canopy analyzer under diffuse-light conditions. Estimated and measured LAI values were compared using the root-mean-square error (RMSE).

Accuracy improvement enabled by CP-PCN

Across both growth stages (bolting and flowering), CP-PCN-based completed point clouds yielded substantially more accurate LAI estimates than incomplete or PoinTr-based completed point clouds (Figure N3).

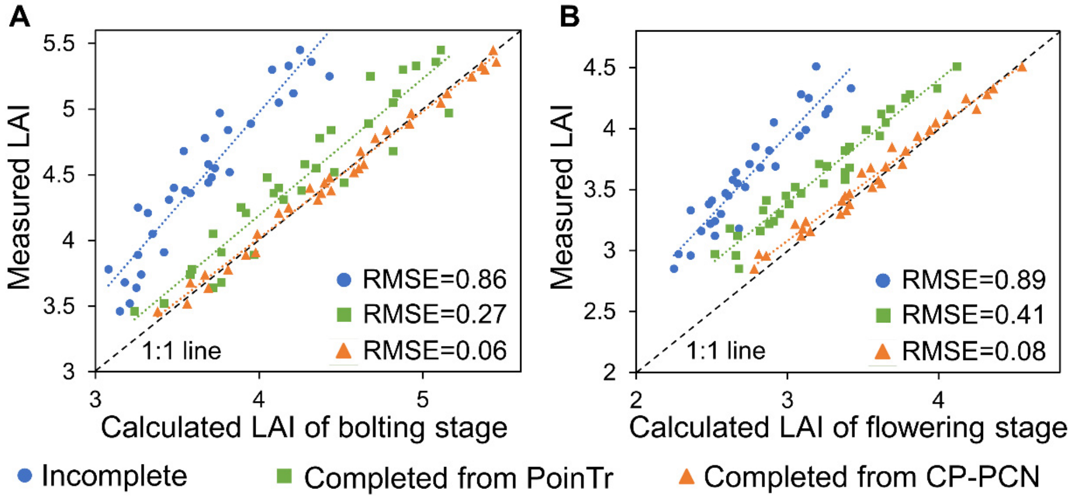

Figure N3. Comparison of LAI estimation accuracy using incomplete, PoinTr-completed, and CP-PCN–completed point clouds at the bolting (**A**) and flowering (**B**) stages.

Incomplete point clouds systematically underestimated LAI, as occluded leaf surfaces were missing. PoinTr-based completion partially reduced missing regions, but introduced scattered noisy points along leaf edges, which caused inconsistent LAI values. In contrast, CP-PCN restored leaf geometry in occluded canopy regions and preserved smooth, anatomically coherent leaf surfaces, leading to the smallest estimation error. These results confirm that

structural completion is not only beneficial for yield-related traits, but also improves the accuracy of physiological traits, demonstrating the broader applicability of CP-PCN for crop phenotyping.

Supplemental Note 8. The detailed architecture of CP-PCN

Multi-resolution encoder

To accommodate the size variation and architectural complexity of rapeseed populations across different growth stages, a multi-resolution feature extraction strategy was conducted in this study. The input point cloud was down-sampled using IFPS to three resolutions, containing N (8192), $N/2$ (4096), and $N/4$ (2048) points, respectively. This hierarchical down-sampling mechanism enabled the network to capture both local and global features, ensuring that architectural details were preserved at multiple scales.

At each resolution level, the dynamic graph convolutional feature extractor (DGCFE) was applied through five layers, where each layer dynamically constructed local graphs to learn spatial relationships and high-level semantic features. Specifically, the feature dimensions of the extracted representations increased progressively from 64, 128, 256, 512, to 1024. To enhance feature aggregation, the output from the last four layers underwent a max pooling operation at each resolution, extracting the most salient feature representations. The pooled feature vectors were then concatenated across all layers, forming a 1920-dimensional latent vector (F). This comprehensive feature representation was subsequently aggregated into a final feature map (M),

which integrated information from all resolutions to facilitate effective downstream point cloud completion.

Dynamic graph convolutional feature extractor

In the CP-PCN model, we designed a DGCFE to extract features from point clouds, leveraging its unique capability to dynamically update neighborhood relationships at each convolutional layer. Specifically, unlike traditional static graph architectures, the DGCFE recalculated k-nearest neighbor (k-NN) relationships based on the evolving feature space at each layer, allowing the network to flexibly adapt to variations in local geometry across layers and enhance spatial feature representation, as shown in Figure 7C.

The DGCFE mechanism was built around the EdgeConv operation, which constructed edge features by considering both the center point and its neighbors. For a given center point p_i and one of its neighboring points p_j, EdgeConv computed an edge feature as follows:

$$h_{ij} = MLP([x_i, x_j - x_i]) \quad (1)$$

where x_i and x_j denoted the feature vectors of points p_i and p_j, respectively, and $[x_i, x_j - x_i]$ represented the concatenation of the center point's features with the relative position vector. A MLP was then applied to transform these concatenated features, capturing complex spatial relationships within each neighborhood.

After computing edge features h_{ij} for each point and its neighbors, a max pooling operation aggregated the edge features, resulting in a unique

representation h_i for the center point p_i:

$$h_i = max_{j=1,\dots,k}(h_{ij}) \quad (2)$$

This pooling operation emphasized the most significant features within each neighborhood, ensuring that the representation remained invariant to the ordering of neighboring points. By repeating this process across multiple layers, the DGCFE progressively refined the neighborhood graph and captured detailed local structures in the point cloud data, yielding a robust and adaptive spatial feature representation. In this study, the size of k was set to 20.

Point pyramid decoder

The PPD was a crucial component of the CP-PCN model, responsible for decoding the high-dimensional feature vector into the missing point cloud. Operating as a hierarchical decoder, the PPD processed the input feature vector at multiple resolutions, ultimately reconstructing the occluded point cloud at three distinct scales. The PPD took the 5760-dimensional final feature vector V as input and generated the missing point cloud of size $M \times 6$, representing the shape of the missing region. The PPD operated as a hierarchical decoder, processing features at different resolutions. The input feature vector was passed through multiple stages to progressively refine the point cloud completion. Initially, the feature vector V was passed through the fully connected layers, resulting in three feature layers: FC_3 (512 neurons), FC_2 (1024 neurons), FC_1 (1920 neurons). Each of these layers was responsible for predicting point cloud structures at different resolutions. At the deepest layer,

FC_3 predicted the primary center points Y_{coarse}, which had a size of $M_1 \times 6$. These primary center points served as the foundation for further refinement. Then, FC_2 predicted the relative coordinates of secondary center points Y_{middle}. Each point in Y_{coarse} acted as a center point to generate M_2 points of Y_{middle}, refining the structure of the predicted point cloud. The process involved "Expand" and "Add" operations, where the size of Y_{middle} became $M_2 \times 6$. Finally, the detailed point cloud Y_{fine} was predicted by FC_1 resulting in the final high-resolution prediction. The generation of Y_{fine} followed the same principle as Y_{middle} but focused on finer structural details, as illustrated in Fig. 2b. The size of Y_{fine} is $M \times 6$, and it was designed to closely match the feature points sampled from the ground truth. By leveraging this hierarchical structure, high-level features extracted from the initial coarse layers influenced the generation of detailed features, thereby minimizing distortion and preserving the fine geometric details of the original missing point cloud. Here, the values of M, M_1, M_2 were set to 8192, 2048, 4096, respectively.

Supplemental Note 9. Design of loss function

The loss function played an essential role in training the CP-PCN model, guiding the optimization of both the feature extraction and point cloud completion processes. It consisted of two key components: multi-stage completion loss and adversarial loss. The completion loss measured the difference between the ground truth missing point cloud Y_{GT} and the predicted point cloud, while the adversarial loss optimized the MRDG and PPD to ensure

that the predicted output appeared more realistic. The size of Y_{GT} was $M \times 6$, which matched Y_{fine}. The commonly used CD, widely applied in point cloud completion research, was adopted as the model's loss function, which was calculated as follows:

$$d_{CD}(S_1, S_2) = \frac{1}{S_1}\sum_{x\in S_1}\min_{y\in S_2}\|x-y\|_2^2 + \frac{1}{S_2}\sum_{y\in S_2}\min_{x\in S_1}\|y-x\|_2^2 \quad (3)$$

where S_1 and S_2 represented two sets of 3D point clouds. CD in Equation (3) measured the average nearest squared distance between the predicted point cloud S_1 and the ground truth point cloud S_2. Since the PPD predicted three point clouds at different resolutions, the multi-stage completion loss was formulated as follows:

$$L_{com} = d_{CD1}\left(Y_{fine}, Y_{GT}\right) + \alpha\, d_{CD2}(Y_{middle}, Y'_{GT}) + 2\alpha\, d_{CD3}(Y_{coarse}, Y''_{GT}) \quad (4)$$

where d_{CD1}, d_{CD2} and d_{CD3} represented the CD at different resolution levels, weighted by the hyperparameter α. The weighting parameter α was progressively updated as the number of training epochs increased. The first term calculated the squared distance between the detailed points Y_{fine} and the ground truth of the missing region Y_{GT}. The second and third terms calculated the squared distance between the primary center points Y_{coarse} and secondary center points Y_{middle} and their corresponding subsampled ground truth Y''_{GT}, Y'_{GT}, respectively. The subsampled ground truth Y''_{GT} and Y'_{GT} were obtained by applying IFPS to extract representative feature points from the missing region and were of sizes $M_1 \times 3$ and $M_2 \times 3$, respectively.

The adversarial loss was inspired by GANs. The generator function was

defined as $F(\quad) = PPD(MRDG())$, where $F: \mathcal{X} \rightarrow \mathcal{Y}'$ mapped the partial input $\mathcal{X}$ to the predicted missing region $\mathcal{Y}'$. The discriminator $D()$ attempted to distinguish between the predicted missing region $\mathcal{Y}'$ and the real missing region $\mathcal{Y}$. The discriminator incorporated both a DGCFE module and an MLP network to compare the predicted point clouds with the ground truth. The structure of the DGCFE remained consistent with its implementation in the MRDG. The MLP network consisted of sequential layers with dimensions [64 – 64 – 128 – 256], where max pooling was applied to the outputs from the last three layers to obtain a compact feature representation. These pooled feature vectors were concatenated to form a 448-dimensional latent vector, which was subsequently passed through fully connected layers [256, 128, 16, 1], and a sigmoid classifier to generate the final discrimination result. The adversarial loss was computed based on the discriminator's performance as follows:

$$L_{adv} = \sum_{1 \le i \le S} \log\big(D(y_i)\big) + \sum_{1 \le i \le S} \log\Big(1 - D\big(F(x_i)\big)\Big) \tag{5}$$

where $x_i \in \mathcal{X}$, $y_i \in \mathcal{Y}, i = 1,2,\ldots,S$, with S representing the dataset size. Both $F()$ and $D()$ were jointly optimized using alternating Adam updates during training.

The final loss function, combining the multi-resolution completion loss and adversarial loss, was formulated as:

$$L = \lambda_{com} L_{com} + \lambda_{adv} L_{adv} \tag{6}$$

where λ_{com} and λ_{adv} were weight hyperparameters satisfying $\lambda_{com} + \lambda_{adv} = 1$. In this study, λ_{com} was set to 0.9.

Supplemental videos

Supplemental video 1. Visualization of completed 3D canopy architectures across multiple viewpoints.

This video presents point cloud completion results generated by CP-PCN at four rapeseed growth stages (seedling, bolting, flowering, silique). The completed population point clouds are shown from multiple viewing angles to illustrate structural integrity, continuity, and recovery of occluded canopy regions.
